# Multifunctional Oxazolone Derivative as an Optical Amplifier, Generator, and Modulator

**DOI:** 10.1021/acs.jpcb.1c08056

**Published:** 2022-02-18

**Authors:** Adam Szukalski, Przemysław Krawczyk, Bouchta Sahraoui, Beata Jędrzejewska

**Affiliations:** † Wroclaw University of Science and Technology, Faculty of Chemistry, Wyb. Wyspiańskiego 27, 50-370 Wrocław, Poland; ‡ Nicolaus Copernicus University, Collegium Medicum, Faculty of Pharmacy, Kurpińskiego 5, 85-950 Bydgoszcz, Poland; § Laboratoire MOLTECH-Anjou, Université d’Angers, UFR Sciences, UMR 6200, CNRS, 2 Bd. Lavoisier, 49045, Angers Cedex, France; ∥ Bydgoszcz University of Science and Technology, Faculty of Chemical Technology and Engineering, Seminaryjna 3, 85-326 Bydgoszcz, Poland

## Abstract

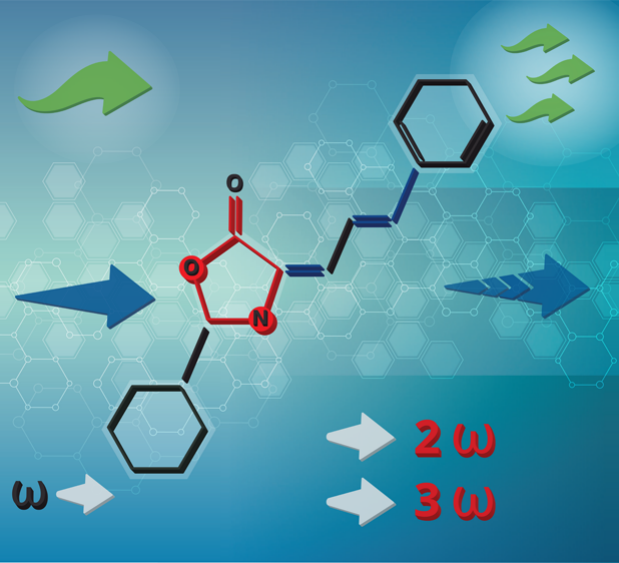

An optical control of many working optoelectronic systems (real-time sensors, optical modulators, light amplifiers, or phase retarders) giving efficient optical gain or remote signal modulation is currently included as scientifically and industrially interesting. In here, an oxazolone derivative as the multifunctional organic system is given in this contribution. The molecule possesses a stilbene group and an oxazolone heteroatomic ring, which implies effective refractive index manipulation and multimode lasing action, respectively. The light modulation is repeatable and stable, also in the hundreds of Hz regime. On the other hand, the amplified optical signal can be easily generated by an external optical pumping source. Thus, signal control is fully available, as is read-in and read-out of the information in real time. Furthermore, this third-order, nonlinear, optical phenomenon using a third harmonic generation technique was also observed. We discovered that only by changing the energy and time regime of the supplied optical signal is the optical or nonlinear optical response observed. Two heteroenergetic molecular states (*trans* (*E*) and *cis* (*Z*)) can efficiently operate in modern multifunctional optoelectronic systems, which can provide and generate an optical signal. Such functionalities are commonly used in all-optical photonic switchers and logic gates and can be utilized in optical-core networks and computers.

## Introduction

Hybrid organic materials (OMs) have attracted attention recently due to many reasons, namely, a broad range of chemical synthesis possibilities, low fabrication costs, versatility in various applications, and their biofriendly character.^1−3^ Both active and passive organic systems were considered due to their interesting linear and nonlinear optical (NLO) functionalities.^4−6^ OMs have found plenty of recommendations in diversified disciplines, such as photonics,^7−9^ organic electronics,^10,11^ and optical networks, including logic gates^12^ or sensing.^2,13^ Therefore, a large number of practical devices are commonly used in the industrial, analytical, or scientific domains. However, two significantly different approaches are currently employed. The materials are supposed to be individually featured for certain utilizations (like in medicine, theranostics is used,^14^ or in material science, where a peculiar polymer, with its unique rheological or structural properties, is used^15^), or their high compatibility and multifunctionality (versatile OMs^16−19^) are expected.

Considering just the light-controlled organic materials, the photochromic polymers should be distinguished.^20,21^ However, macromolecules can also serve just as the inert and passive branched matrix. Then, a low-molecular compound acts as the active component, and its properties give the final multiple material functionality.^22^ There are many examples of such working organic systems. Interestingly, in the field of spectroscopy and nonlinear optics, such OM utilization was observed recently. Multiarmed structures with various symmetries characterized efficient two-photon absorption (TPA) and a photorefraction phenomena.^23^ Depending on the arm type in such synthesized molecules, their NLO properties are different. Intriguingly, the centrosymmetric NLO active system did not give any of photorefraction effect. Such an investigation approach leads to the construction of solid-state optical limiting devices and TPA-excited photorefractive systems. Furthermore, an efficient third-order NLO active system (polyoxometalates functionalized by porphyrin-based Schiff base), also characterizing catalytic properties, was recently presented in the literature.^24^

Oxazolones have been of great interest in medicine since the 1980s.^25−27^ They are used in chemically induced protocol models of intestinal inflammation.^28^ However, the oxazolones (together with pyrazolones and pyrazolines) were also investigated in the context of their spectroscopic properties.^29^ There are few contributions in the literature presenting the oxazolone synthesis route and the basic spectroscopic properties (absorption and emission spectra).^30^ Giving an example, the experimental and theoretical studies were performed to describe the influence of solvent polarity on the spectroscopic features of the oxazolone derivative.^31,32^ Due to the aforementioned properties, the oxazolones found an application in sensing (in particular, in precise Fe^3+^ ion detection^33^) or biosensing (acetylcholine reversible detector^34^). It was found that oxazolone derivatives (as the green fluorescent proteins) characterize photophysical properties quite similar to the bioluminescent organisms existing in nature.^35^ Importantly, the NLO properties also were determined in the group of oxazolones. A significant TPA phenomenon,^36−38^ or a third-order nonlinear optical response (investigated in the degenerate four wave mixing experimental setup),^39^ was reported.

Here, we present a low-molecular multifunctional oxazolone derivative characterizing efficient optical and nonlinear optical properties. The introduced compound can serve as a productive light amplifier, generator (of higher harmonics of light), and electromagnetic wave modulator in the submicroseconds time scale. To the best of our knowledge, it is the first report on the oxazolone derivative being such a comprehensive organic-based hybrid material. An acquired optical gain profile indicates that the oxazolone derivative is a highly effective laser dye. Furthermore, it was experimentally proven that the hybrid polymeric system doped with the investigated molecule provides a three times higher third-order NLO signal than the reference material. Finally, an all-optical switching phenomenon was observed, giving a stable and reversible nonlinear optical response when the pump channel is modulated in the range of hundreds of Hz.

## Experimental Section

### Synthesis and Structural Analysis

All solvents (spectroscopic grade, Table S1 in the Supporting Information (SI)), hippuric acid, cinnamaldehyde, and acetic anhydride were purchased from either Aldrich Chemical Co. or Chemat Co. Poland, and they were used without further purification.

**Figure 1:**
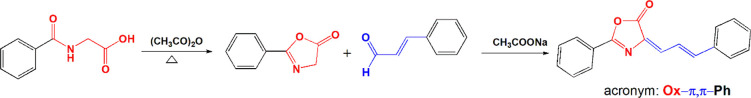
Route for the synthesis of 4-(3′-phenyl-2′-propenylidene)-phenylooxazol-5(4*H*)-one (its acronym, Ox-π,π-Ph, with marked significant regions; red: oxazolone ring; blue: two conjugated π-bonds creating stilbene groups in the final product).

The oxazolone dye, 4-(3′-phenyl-2′-propenylidene)-phenylooxazol-5(4*H*)-one, was synthesized in our laboratory according to the method described by Luna et al.^40^ and Khan et al.^41^ The route for the synthesis is presented in Figure 1, and details are given in the SI.

### Characterization

#### Spectral Measurements of the Solutions

Electronic absorption spectra for an approximately 10^–5^ M dye solution in solvent of different polarities were recorded at room temperature on a Shimadzu UV–vis Multispec-1501 spectrophotometer. Emission spectra were obtained from a Hitachi F-7100 fluorescence spectrophotometer. The solution concentration was about 10^–6^ M.

The fluorescence quantum yield (FQY) was calculated from eq 1:^42^
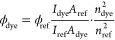
1where ϕ_dye_ and ϕ_ref_ are the FQY of the dye and the reference, respectively; *I*_dye_ and *I*_ref_ are the integrated intensities (areas) of the sample and reference spectra, respectively (in units of photons); *n*_dye_ and *n*_ref_ are the refractive indexes of the solvents used for the dye and the reference, respectively. The absorbances (*A*) of both the dye and the reference solution at an excitation wavelength (380–404 nm) were about 0.1. Coumarin 153 in ethanol (ϕ_ref_ = 0.38^42^) was used as the reference. The solvent effect on the spectral properties of the tested dye was analyzed, applying a multilinear correlation based on the four-parameter Catalán^43^ solvent scale (Table S1 in the SI).

#### Photostability

The photostability experiments were carried out in a quartz cuvette with dimensions of 4 × 1 × 1 cm that was placed in a horizontal position to ensure complete absorption of light (the optical path length = 4 cm). An approximately 1.5 × 10^–5^ M solution of the dye in ethyl acetate was stirred and irradiated through the bottom wall of the cuvette with a diode-pumped solid state (DPSS, Shanghai Dream Lasers) laser light at 408 nm (light power of 35 mW). The bleaching of the dye at the absorption peak was monitored as a function of time. Therefore, the absorption spectra were recorded at different times during the irradiation.

#### Computational Details

All geometrical parameters of the investigated molecules in their ground (S_GS_) and excited states (S_CT_) were calculated using density functional theory (DFT) approach implemented in Gaussian 09 program package^44^ with TIGHT threshold option and PBE0/6-311++G(d,p) basis set. To verify that all the structures correspond to the minima on the potential energy surface, an analysis of Hessians was performed. The electronic properties were characterized by computations of the vertical absorption and emission spectra, which were obtained using the time-dependent density functional theory (TDDFT/PBE0)^45^ and by including the state-specific (SS) corrected linear response (cLR) approach.^46^ All spectroscopic calculations were performed using the standard-hybrid PBE0^47,48^ functional. The dipole moments and polarities of the charge-transfer state (CT) were evaluated by numerical differentiation of the excitation energies (*E*) in the presence of an electric field *F* of 0.001 au strength:
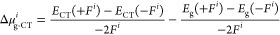
2where *i* stands for the Cartesian component of the dipole moment difference. The isotropic average polarizability (α), polarizability anisotropy (Δα), and first-order hyperpolarizability (β_vec_) were determined based on the Gaussian 09 program and defined as
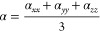
3
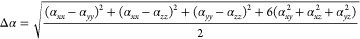
4
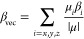
5where β_*i*_ (*i* = *x*, *y*, *z*) is given by .

The density differences were obtained at the PBE0/6-311++G(d,p) level and are represented with a contour threshold of 0.02 au. In the graphs (e) and (f) in Figure 4, the blue (purple) zones indicate density decrease (increase) upon electronic transition. The charge transfer parameters, namely, the charge-transfer distance (*D*_CT_) and the amount of transferred charge (*q*_CT_), have been determined following a Le Bahers’ procedure.^49^ The solvent effect on the linear and nonlinear optical properties has been taken into account using the Integral Equation Formalism for the Polarizable Continuum Model (IEF-PCM).^50,51^

#### Solid State Spectroscopy

For the needs of the spectroscopic studies of the Ox-π,π-Ph chromophore in the solid state, functionalized thin polymeric films were prepared. A commercially available polymer–poly(methyl methacrylate) (PMMA) in the powder form (*M*_w_ = 120 kDa) served as the branched matrix (host material). At first, a mixture of the dye (guest) and polymer (host), using dichloromethane (DCM) solvent, was prepared. The dye constituted a 2% dry mass of polymer. After mixing at room temperature, a homogeneous solution (400 μL) was deposited separately onto a silica glass plate by the drop casting (d-c) and spin-coating (s-c) techniques (dependent on the future destination), respectively. The d-c thin film was formed together with the DCM atmosphere over the next 2 days. Such a prepared layer (drop-casted) was investigated using a spectrophotometer (Hitachi U-1900) and a spectrofluorometer (Hitachi Fluoromax-4). The spin-coated layer was stored in the darks after being prepared at room temperature and in an ambient atmosphere. Additionally, the d-c and s-c film thickness values were measured by a profilometer (Dektak-3), and they are equal to 14.08 and 1.92 μm, respectively. The light amplification studies, but also the following second and third-order NLO effects (optical Kerr effect and second harmonic generation), require a significant active system’s volume in order to (*i*) effectively induce the lasing action (inverted *Boltzmann* distribution generation), (*ii*) generate optical birefringence (induce molecular reorientation), or (*iii*) break symmetry using the corona poling technique toward noncentrosymmetry. For the purposes of the third harmonic generation phenomenon investigation, where it was previously required, the spin-coated layer was utilized (in that way any of reabsorption processes during the experiment were minimized).

The light amplification studies were performed using an experimental setup, where a nanosecond pulsed laser source (Surelite II, Continuum, *f* = 10 Hz, *t* = 6 ns, λ = 355 nm) was utilized (Figure S1(a)). However, the fundamental laser beam wavelength was switched at 420 nm thanks to the use of optical parametric oscillator (OPO, Horizon, Continuum). Such an optical adjustment is allowed to develop the laser setup to the investigated system (the incident laser beam was in resonance with the laser dye, which provides its efficient Boltzmann redistribution conversion, leading to the radiative nature of energy dissipation).^52^ The linearly polarized incident laser spot (in the typical round shape) was obtained after spatial conversion thanks to the mounted lens system characterized the stripe-shape view. In this manner, the excitation laser beam hit the sample in a controllable way, localized close to the edge of substrate. The Variable Stripe Length (VSL) method^53^ was used in order to investigate an optical gain profile in the active system (Figure S1(b)). Thanks to the linear shape of the excited area, the output light amplification ray was directed toward the sample’s edge, which resulted in its efficiency maximization. The other directions of LA were laden according to light diffusion or reabsorption phenomena (Figure S1(c)). The output signal was collected by a high resolution spectrometer (Shamrock 163, ANDOR Solis) equipped with an external 200 μm optical fiber. The acquired emission characterized the optical gain profile, which is typical for a multimode random lasing (RL).^52,53^ Since the light enhancement is represented by the existence of multiple laser modes, the Power Fourier Transform (PFT) was helpful with the optical resonator dimension estimation.^54,55^ The optical resonators can be defined in two various models, namely, in the Whispering Gallery Modes (WGM)^56^ (eq 6) or in Fabry–Perot ones, respectively^57^ (eq 7):
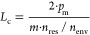
6
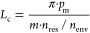
7where *m* and *p*_m_ denote the ordering and magnitude of the analyzed PFT signals (the emission wavelength was expressed by a *k* factor (1/μm)). The *p*_m_ number should give periodically and proportionally increasing values with the lower intensity, whereas the *n*_res_ and *n*_env_ represent the refractive index value of the active system and the experimental environment, respectively. In our case, the *n*_res_ was equal to 1.5031, which corresponds to the PMMA refractive index value at 420 nm.^58^ Subsequently, the *n*_env_ comes from the air (1.0003).^59^ Thanks to the additional RL spectra output analysis (average of 20 collected spectra), like emission integration or the full width at half-maximum (fwhm) parameter, the energy threshold estimation was performed.

#### Nonlinear Optics

Optical Kerr Effect (OKE) and third harmonic generation phenomena represent third-order NLO effects. It means that the active medium symmetry does not influence so far on the output results, like in the case of the second-order phenomena, for example, second-harmonic generation.^60,61^ The OKE phenomenon is also connected with a molecular reorientation process, or all-optical switching, where the NLO medium is susceptible to the influence of a high energy laser light source, which complies with the chromophore’s absorption resonance. In such a way, the conformational conversion induced by the electromagnetic wave reflects in the optical anisotropy generation. It is strictly involved with molecular movements and transformations between lower in energy and stable states (usually, *trans* (or *E*) conformers) and higher in energy, metastable forms (*cis* (or *Z*) ones). Due to the photons absorption probability (*P* = cos^2^(α), where α denotes an angle between the input laser polarization direction and its molecular equivalent), the molecules are oriented in a particular way, giving as the result the refractive index anisotropy (photoinduced birefringence, Δ*n* (*I*_pump_,*t*)).^60,61^ The just-mentioned interaction between laser light and the NLO active medium is responsible for the refractive index indicatrix deformation, which reflects on the second, nonlinear refractive index (*n*_2_) generation, according to the following equation:
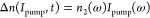
8

Optical anisotropy generation is strictly involved with phase change (Δφ), which is consequently related with pump laser beam intensity (*I*_pump_) over time Δφ(*I*_pump_, *t*)*a*:
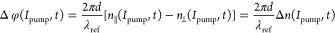
9where *d* denotes the sample thickness, λ_ref_ is the monitoring laser beam wavelength (called also reference or reading beam), *n*_∥_(*I*_pump_, *t*) and *n*_⊥_(*I*_pump_, *t*) refer to the parallel and perpendicular components of the NLO chromophore refractive index (coming from its indicatrix) with respect to the polarization direction of the pump; Δ*n*(*I*_pump_, *t*) means the photoinduced birefringence in *I*_pump_ and time and function, respectively.^60,61^ After a few simple transformations of eq 9, a clear relation can be distinguished between NLO and the experimental setup parameters, which is shown below:^60,61^
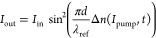
10where *I*_out_ and *I*_in_ denote to the reference laser beam intensity behind the analyzer and before the polarizer (the initial one), respectively. The geometrical component (sin^2^(2α)) from eq 10 was simplified and is equal to 1, because the pump laser polarization configuration was aligned at 45° due to the reference one.

Second and third harmonic generations (SHG and THG, respectively) represent second and third-order NLO effects,^60,61^ accordingly. Only noncentrosymmetric NLO active media can efficiently give SHG signal. The other ones, due to the theoretical reasons described elsewhere, cannot (it stems from the annihilation of the second-order susceptibility).^60,61^ From this reason, the centrosymmetric systems (i.e., thin polymeric films doped with NLO chromophores) need to undergo a special thermoelectric treatment (corona-poling).^62,63^ Drop-casted thin film is at first (1) heated up slightly above the glass temperature (this parameter corresponds directly to the utilized polymer); (2) controlled high voltage (typically 5 or 10 kV) is then applied to two opposite plates or other electrical conductors (needles/wires, oriented perpendicular to the sample’s surface), but at the same moment, the heating system is switched off; (3) thermal relaxation with the electric current ordering is ongoing until the thin film again reaches the room-temperature conditions. Usually, after a few hours, the thin polymeric film doped with polar low-molecular chromophores is solidified and the characteristic symmetry is broken, and in that shape, it is able to give the SHG signal. THG experiments do not require any particular conditions related with molecular symmetry, so that step can be skipped during sample preparation. The laser setup, where Maker fringes are generated and investigated via a comparable method, is already described in detail elsewhere in the literature.^64−66^ Briefly, a fundamental beam, provided by a picosecond pulsed laser equipped with an electronic system that controls and triggers all signals, is focalized on the sample surface. The active medium in the shape of film or crystal is then twisted on the rotational stage, typically from −80° up to 80° (or −60° to 60°).^64−66^ Before and behind the sample there is a crossed-polarizer system that can provide additional information about spatial SHG properties. Finally, generated NLO response (SHG or THG) is delivered to the photomultiplier equipped with a dedicated filter (to separate fundamental and generated laser modes). The SHG signal can be analyzed according to the Lee model (eq 11):^67^
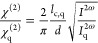
11where χ^(2)^ and χ_q_^(2)^ refer to the second-order nonlinear optical susceptibility value for the investigated and reference material, which is quartz in our case (),^68^ respectively. Subsequently, the *l*_c_,_q_ defines the quartz coherence length (eq 12), and *d* refers to the thickness of the investigated system. The *I*^2ω^ and *I*_q_^2ω^ refer to the measured second harmonic signal intensity for the sample and quartz, respectively.
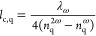
12where λ_ω_ denotes the fundamental beam wavelength and *n*_q_^ω^ and *n*_q_^2ω^ are the refractive indices values for 1064 nm (1.534) and 532 nm (1.547), respectively.^69^

While the third harmonic of light generation phenomenon can be estimated in a reliable way thanks to the Kubodera and Kobayashi theoretical model (eq 13).^70^ In this case, a silica slab serves as the reference material with a known third-order NLO susceptibility value ( at 1064 nm^71,72^). Whereas, according to eq 13, it is possible to define the considered third-order NLO parameter for the investigated medium as follows:
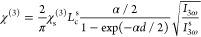
13where α and *d* define the absorption coefficient at the third harmonic of light (355 nm) and sample thickness, accordingly (such approach is correct, when transmittance is less than 0.9). The *I*_3ω_ and *I*_3ω_^s^ refer to the THG signal intensity collected for the investigated material and silica, accordingly, while the *L*_c_^s^ parameter is the coherence length of silica, defined as follows (eq 14):
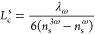
14where *n*_s_^3ω^ and *n*_s_^ω^ describe the refractive indices for the silica at 355 nm (1.4761) and 1064 nm (1.4496), respectively.^73,74^

## Results and Discussion

### Synthetic Procedure and Molecular Design

The 4-(3′-phenyl-2′-propenylidene)-phenylooxazol-5(4*H*)-one (Ox-π,π-Ph) was synthesized using a method described earlier,^40,41^ taking hippuric acid as the substrate in the reaction with cinnamaldehyde in the presence of acetic anhydride and anhydrous sodium acetate. The detailed synthetic route with NMR and IR spectra analysis is presented in the SI.

The design concept of the compound is based on the 5-(4*H*)-oxazolone ring, which is the precursor of the green fluorescent proteins chromophore and constitutes an electron-withdrawing part of the molecule. From both sides of the heterocycle ring (at 2 and 4 positions) there are aromatic rings that create terminal moieties with an electron-donating character. They have the net effect of increasing electron density in the molecule. One of the phenyl rings is bonded to the 5-(4*H*)-oxazolone by two π-conjugated double bonds that are responsible for high molecular flexibility. Thus, its structure, especially the presence of methine groups and an oxazolone ring, will have an influence on the photophysical and photochemical properties of the dye due to the possibility of conformational transformations (i.e., *E-Z-E*) or partial molecular rotation.

**Figure 2:**
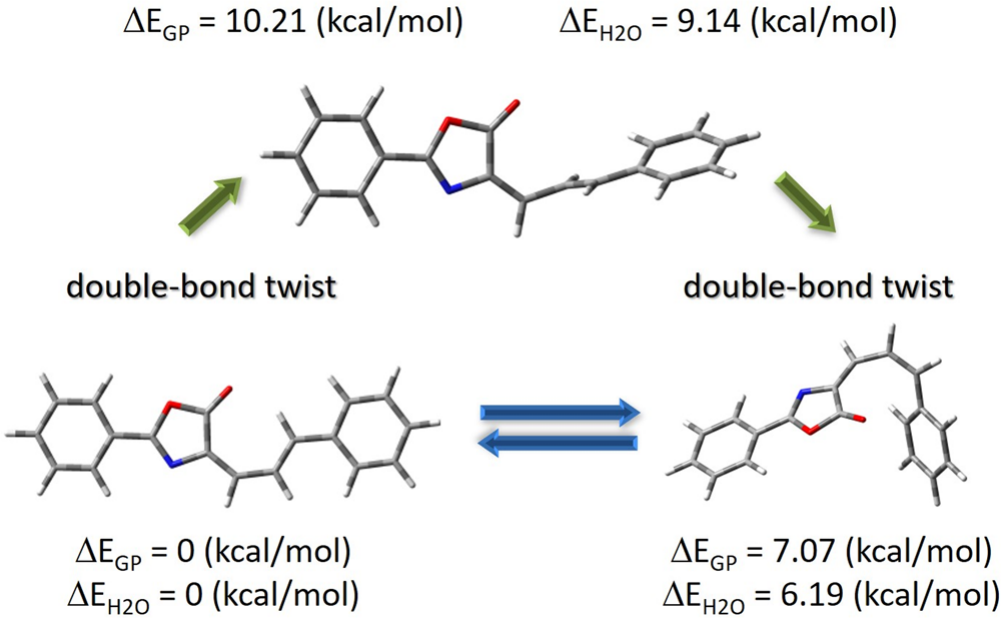
Optimized structures of the Ox-π,π-Ph isomers. Transition state geometry obtained from the QST3 approximation (wb97xd/TZVP).

Since the dye may exist in different isomers, the DFT equilibrium geometries of the compound were optimized both in the gas phase and in water environments. The optimized structures of the most stable *E* and *Z* conformers of the Ox-π,π-Ph molecule are presented in Figure 2, and the data are collected in Tables S2 and S3 (atom numbering is shown in Figure S2).

There are several local minima on the Potential Energy Surface (PES); however, in this study, a detailed analysis of the linear and nonlinear optical properties is presented only for the minima characterizing the lowest energy of the *E* and *Z* isomers. The *Z* conformer is 7 kcal/mol higher in energy than the *E* one. The estimated barrier of *E* → *Z* isomerization amounts to slightly over 10 kcal/mol in GP, whereas the Δ*E* decreases to 9.14 kcal/mol in a water environment. According to the relaxed scan of the PES along two torsion angles (Figure S3), based on the wB97xd functional,^75^ it results mainly from the change in the ΘC6=C8—C9=C10 angle, that is, from 0.00° to 94.36° during the forming of the TS structure. In the case of the ΘC8—C9=C10—C11 angle, the change is from 180.00° to 178.78°. For the discussed derivative, the path of formation of the TS structure follows the rotation of the C=C double bond. At the point of the π-bond rupture, a zwitterionic intermediate forms, generating an anionic carbon center and a corresponding carbocation. The zwitterion contains a C—C single bond, thus, permitting the complete rotation. Another important feature is the development of the C=C double bond character within the transition state.^76^ The analysis of structural parameters revealed a high sensitivity to the environmental changes for the bond lengths and dihedral angles of Ox-π,π-Ph, as well as to those occurring during photoexcitation to the first singlet excited state (S_CT_). The most important selected geometry parameters for these conformers are listed in Tables S2–S4. Primarily, during the reduction of the C1—C2 bond length as the solvent polarity increases, the approach of the phenyl ring to oxazolone can be observed. At the same time, the C4=O5 double bond is elongated, and the O5 oxygen atom approaches the O3 atom. This is the result of a decrease in the ΘO3—C4—O5 angle with a simultaneous increase in the ΘO5—C4—C6 and ΘO5—C4—C6—C8 angles. The environment polarity also affects the π-electron bridge. The C6=C8 and C9=C10 double bonds are elongated with a simultaneous reduction of the C8—C9 and C10—C11 single bonds. The length of the sternum also changes. The angles ΘC6=C8—C9 and C9=C10—C11 are elongated, while the C8—C9=C10 angle is slightly shortened. The solvent polarity does not affect the C12—O14 bond, however, it promotes the extension of O14—C15.

According to the experimental results, the compound showed good photochemical stability under the measurement conditions (Figure S4), since visible light irradiation of the oxazolone dye in ethyl acetate does not cause distinct changes in the electronic absorption spectra. After 85 min of irradiation with a DPSS laser (408 nm) with a light intensity of 35 mW at room temperature, the intensity of the long-wavelength absorption band decreased only 5% without any changes in its position.

### UV–Vis Absorption and Fluorescence Spectra in Solutions

**Figure 3:**
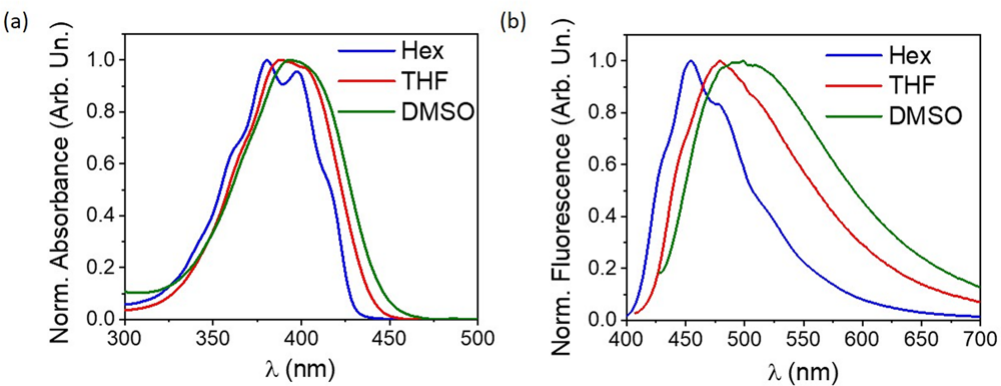
Normalized electronic absorption (a) and fluorescence (b) spectra of Ox-π,π-Ph in *n*-hexane (Hex), tetrahydrofuran (THF), and dimethyl sulfoxide (DMSO).

The solvent effect on the UV–vis absorption and fluorescence spectra of the Ox-π,π-Ph studied in various organic solvents at room temperature is shown in Figure 3, whereas the corresponding photophysical data are collected in Table 1.

**Table 1:** Main Photophysical Parameters^a^ of Ox-π,π-Ph

solvent	λ_max_^Ab^	ε	fwhm^Ab^	λ_max_^Fl^	fwhm^Fl^	Δν	ϕ_Fl_
Hex	381	64000	3892.7	454	3925.7	4220	0.256
TMP	381	58600	3937.1	453	3986.9	4172	0.279
MCH	382	63300	3895.1	455	3911.9	4200	0.285
Bu_2_O	383	52000	3898.3	461	4116.3	4418	0.247
Et_2_O	382.5	58100	4041.5	466	4573.6	4685	0.065
EtOAc	386	60600	3920.4	476	5050.4	4898	0.068
THF	388	58500	3931.3	479	4707.1	4896	0.069
MeAc	384	51700	4254.2	480	5383.9	5208	0.039
MeCN	384.5	55500	4008.0	482	5877.2	5261	0.026
DMF	389	54100	3994.4	488	5375.5	5215	0.033
DMSO	392	51400	3944.6	492	5078.0	5185	0.038
1-BuOH	385.5	54470	3975.0	472	4432.2	4754	0.066
2-PrOH	386.5	51800	3963.1	470	4573.6	4597	0.057
1-PrOH	385.5	50800	4036.5	472	4560.7	4754	0.066
EtOH	386.5	49400	3962.7	477	4738.0	4909	0.053
MeOH	385.5	50600	4270.0	480	5364.5	5107	0.024

a Absorption (λ_max_^Ab^, nm), maximum extinction coefficient (ε, 10^4^ M^–1^ cm^–1^), fluorescence maxima (λ_max_^Fl^, nm), full width at half-maximum (fwhm; cm^–1^), Stokes shift (Δν, cm^–1^), and fluorescence quantum yield (ϕ_Fl_, %).

The UV–vis absorption spectra of Ox-π,π-Ph (Figures 3a and S5(a)) show one main band in which the position, intensity, and shape change with the solvent used. In aprotic nonpolar solvents, the absorption maximum is localized at ∼380 nm, and the spectra reveal a clear vibrational fine structure. With increasing solvent polarity, the first (S_0_ → S_1_) band is slightly red-shifted and reaches a maximum at 392 nm in DMSO. It is almost structureless. In the case of polar protic solvents, the dye shows a limited absorption band maxima diversity with a maximum located close to 386 nm. Additionally, the long-wavelength band of the dye in nonpolar aprotic solvents is about 15% more intense (the higher ε) than the one recorded in alcohols. In general, the molar extinction coefficient has a large value (ε ∼ 6.4 × 10^4^ to 5.06 × 10^4^ M^–1^ cm^–1^) and slightly decreases with increasing solvent polarity. All of this indicates that the absorption originates due to the π → π* transition of a charge-transfer character.

Similarly, to the electronic absorption spectra, in nonpolar solvents, such as hexane and methylcyclohexane, the fluorescence spectra of Ox-π,π-Ph exhibits fine structure. However, when the polarity of the solvents increased, the fluorescence bands are structureless and red-shifted (Figures 3b and S5(b)). For example, in hexane, the fluorescence emission bands of Ox-π,π-Ph exhibit a vibrational structure and appear at 454 nm, whereas Ox-π,π-Ph is structureless with band maximum at 492 nm in DMSO. Red-shifting of the fluorescence maxima in the case of alcohols reaches only 8 nm, which may be due to the hydrogen-bonding ability of the solvents that influences the stabilization of the electronic states of the dye in a different way.

However, the observed positive solvatochromism, large Stokes shifts (such as 5185 cm^–1^ in DMSO), and broadening of the emission band, as well as the higher sensitivity of fluorescence spectra (with respect to absorption) to the dielectric constant of the environment, hint at an intramolecular charge transfer (ICT) character in the compound, which is consistent with its molecular structure.^77−80^

**Figure 4:**
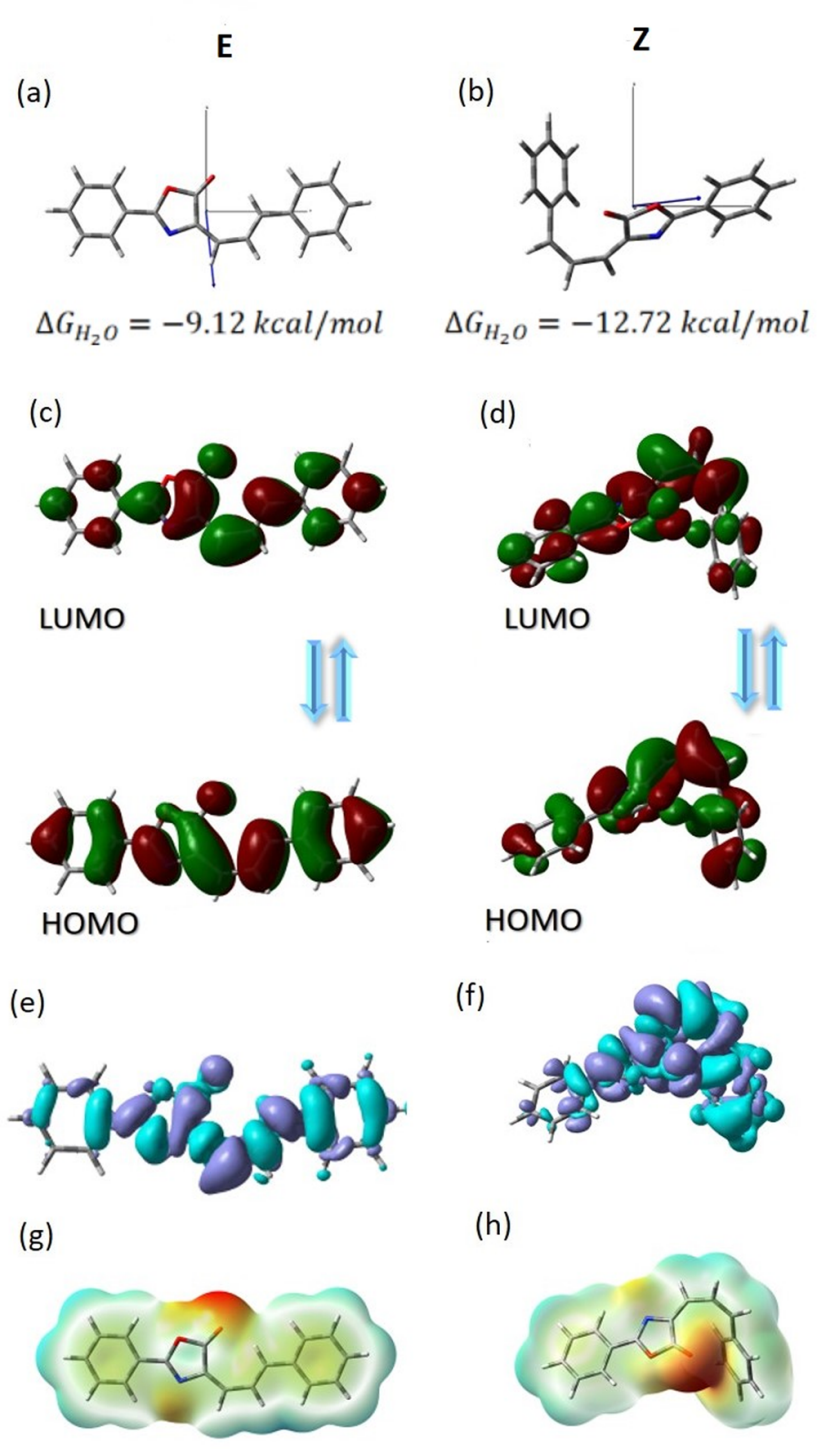
Physicochemical properties of the *E* (left column) and *Z* (right column) Ox-π,π-Ph isomers. Δ*G* energy in a water environment and orientation of the dipole moment vectors for the ground state (a, b); HOMO–LUMO and their gap energy in gas (3.2488 and 3.6701 eV) and water (3.2292 and 3.6233 eV) environments (c, d); density difference for gas (*D*_CT_ = 1.027 Å, *q*_CT_ = 0.323*e*, and *D*_CT_ = 1.505 Å, *q*_CT_ = 0.505*e*) and water (*D*_CT_ = 1.216 Å, *q*_CT_ = 0.355*e*, and *D*_CT_ = 1.685 Å, *q*_CT_ = 0.504*e*) environments (e, f); and MEP in gas (±0.04640 and ±0.05160) and water (±0.05462 and ±0.06279) phases for *E* and *Z* isomers, respectively.

According to the computational calculations, the charge-transfer (CT) excitation for both conformers (*E* and *Z*) corresponds basically to the HOMO → LUMO transition (Figure 4, graphs c and d). HOMO electrons are delocalized on the entire surface of the compound, while the LUMO electrons are mostly on the π-electron bridge. The transfer of electrons from the benzene rings, as a donor group, toward oxazolone and the carbon bridge is observed. This indicates that the lowest-lying excited state can be assigned as a π–π* transition mixed with an intramolecular charge-transfer process. Isomerization does not change the distribution of both frontier molecular orbitals.

There is also no significant change in the value of the energy separation between the HOMO → LUMO orbitals (*E*_GAP_). The Δ*E*_GAP_^*E*–*Z*^ difference is equal to 0.42 eV in the gas phase, and it is reduced to 0.39 eV in the aqueous phase. Although the *E* GAP is slightly decreased in the function of solvent polarity (Table S5), the investigated conformers are characterized by a low value of chemical hardness (η) and should be treated as the soft molecules with very high reactivity. Moreover, the calculated electronegativity (χ), which is greater than 4.0 eV, indicates an easy formation of covalent bonds during various chemical processes. To predict reactive sites for nucleophilic (positive, blue regions) and electrophilic (negative, red and yellow regions) attack of the investigated conformers, the Molecular Electrostatic Potential (MEP) surfaces were calculated (Figure 4, graphs g and h). The most negative region is located on the oxygen atom O5 in the oxazolone ring (*V*(*r*) > −0.04 au). The maximum positive site is localized on the π-electron bridge.

According to the experimental data, analyzed dye is characterized by one strong absorption and emission band, corresponding to the HOMO → LUMO photoexcitation, which is described as π–π* transition. However, non-negligible contributions from the other orbitals may occur. For this reason, the density variation upon photoexcitation (Δρ(*r*)), calculated for the first electronic transition is graphically depicted in Figure 4, graphs e and f, and Table S6. For all compounds, the Δρ(*r*) plots show that the density depletion zones (marked in blue) are mostly delocalized on the benzene rings. The disappearance places on both rings are almost equally acceptable. In turn, for the molecule, the regions of density increment (purple) are mostly localized on the carbon bridge and oxazolone ring. At the same time, the solvent polarity does not significantly change in the location of these regions. This is reflected in the amount of transferred charge (*q*_CT_) and charge-transfer distance (*D*_CT_). Both *q*_CT_ and *D*_CT_ values are large for the *Z* isomer. The Δρ_CT_^*Z*–*E*^ is equal to 0.15 au (Table S6), while the Δ*D*_CT_^*Z*–*E*^ is 0.5 Å in less polar solvents and 0.45 Å in more polar ones. The *D*_CT_ indicates the CT character and confirms the contributions from the HOMO → LUMO transition, although minor contributions from other orbitals should be expected.

The excitation and observation wavelengths do not significantly influence the position and shape of both the fluorescence and fluorescence excitation spectra (Figure S6). However, a high discrepancy between the absorption and the fluorescence excitation spectra occurs when the dye concentration increases (Figure S7). For high compound loadings (100 μM), the main absorption band disappears with the simultaneous appearance of two bands in the short and long wavelength regions of the spectrum, respectively. The increase of the Ox-π,π-Ph concentration up to 1 mM results in an intense, narrow band centered at 439 nm. This behavior is typical for dyes whose molecules self-associate to form *J*-aggregates.^81^ The same relation was obtained by comparing the theoretically determined absorption maxima band for the monomer and the optimized dimer (Figure S8). The absorption bands of the *J*-aggregates are shifted toward longer wavelengths. In solutions with specific interactions of the test compound with solvent molecules (MeCN and water), the bathochromic shift is smaller (approximately 35 nm). In environments where these interactions are absent (DMF and DMSO), the solvatochromic shift is greater and amounts to about 46 nm. Upon increasing the concentration, the fluorescence spectra becomes broad, narrower, and moves toward longer wavelengths, which also implies that aggregates are formed (Figure S7(b)).^81^

The Ox-π,π-Ph demonstrates a significant variability in the fluorescence quantum yields (FQY) in different solvents. As can be seen in Table 1, the FQY returned the higher values (ϕ_Fl_ of 25.6%, 27.9%, 28.5%, and 24.7%, respectively) in nonpolar aprotic solvents, like Hex, TMP, MCH, and Bu_2_O, than in other solvents for which it is about an order of magnitude smaller. The decrease in the emission intensity with increasing solvent dielectric constant is typical for dyes exhibiting a large charge separation in their excited state.^82,83^ The lack of dual emission in polar solvents suggests that the ICT state of Ox-π,π-Ph undergoes a nonradiative relaxation to the ground state. Similar results were observed for aminated derivatives of the GFP chromophore^84^ and for some arylacetylenes.^85^ Thus, the vibrations and rotations around the single and double bonds separating the oxazolone scaffold and the side phenyl rings and the charge-transfer (CT)^86^ interaction are likely to quench the emission of the dye in solution.^87−89^ The CT excited state is a covalent (bonding) state with an opposing charge and bond localization relative to the ground state (GS). Moreover, a low FQY in polar protic solvents may relate to the site-selective hydrogen-bonding-induced fluorescence quenching effect proposed by Yang et al.^90^

The specific and nonspecific interactions^91,92^ between the dye and solvent were analyzed by means of the Linear Solvation Energy Relationships (LSERs) concept using Multiple Linear Regression Analysis (MLRA) based on the four parameters scale proposed by Catalán et al.^43^

The solvatochromic behavior of Ox-π,π-Ph is in good agreement with the Catalán model, which correlates the spectral shift ν of the solute with the solvent parameters that are responsible for the polarizability (SP), dipolarity (SdP), acidity (SA), and basicity (SB) properties of the latter:^43,93,94^
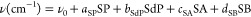
15

Multivariable regression of the absorption and emission as well as Stokes shift and FQY data of Ox-π,π-Ph are summarized in Table 2. The applied Catalán linear regression analysis resulted in good fits for all data for the oxazolone dye with correlation coefficients of 0.889, 0.938, 0.876, and 0.902 for absorption, fluorescence, Stokes shift, and FQY, respectively. Besides FQY, these data correlate even better with the applied model when protic solvents (alcohols) are excluded. The standard errors are equal to 0.918, 0.988, and 0.998, respectively (see Table S7).

**Table 2:** Solvatochromic Spectral and FQY Parameters of the Ox-π,π-Ph Dye^a^

ν	ν_Ab_	ν_Fl_	Δν^SS^	ϕ_Fl_
ν_0_	27671 ± 243	23216 ± 470	4454 ± 476	0.04789 ± 0.10845
*a*_SP_	–(2247 ± 389)	–(1955 ± 752)	–(292 ± 763)	0.35721 ± 0.17372
	79.7%	53.5%	13.0%	47.2%
*b*_SdP_	–(163 ± 69)	–(1360 ± 133)	1198 ± 135	–(0.25225 ± 0.03072)
	5.8%	37.2%	53.5%	33.3%
*c*_SA_	–(225 ± 125)	222 ± 242	–(447 ± 245)	0.0688 ± 0.0559
	8.0%	6.1%	20.0%	9.1%
*d*_SB_	–(184 ± 97)	117 ± 188	–(301 ± 191)	–(0.07857 ± 0.04339)
	6.5%	3.2%	13.5%	10.4%
*R*^2^	0.889	0.938	0.876	0.9016

a The corresponding coefficients were calculated using multivariable linear regression analysis, applying the Catalán approach. *R*^2^ is the correlation coefficient.

Based on the contribution percentages of different polarity parameters, solvent polarizability (SP) was found to have the highest influence on the positions of the absorption band, whereas the solvent dipolarity, acidity, and basicity reveal low contributions. The negative sign of all Catalán coefficients in the absorption process indicates a bathochromic shift with increasing solvent polarizability (SP), dipolarity (SdP), acidity (SA), and basicity (SB), thus, the ground state’s energy level increases through these parameters. Additionally, since the absorption band position is mainly controlled by nonspecific interactions resulting from solvent polarizability (SP), the absorption arises from a polarized π–π* transition.^29^

Excitation led only to insignificant changes in the SA and SB terms, which indicates similar acidity and basicity of the ground and excited states^87^ of the Ox-π,π-Ph dye. On the other hand, the polarizability is reduced (a decrease in the SP coefficient) accompanied by an increase in SdP. Thus, solvent’s polarizability and dipolarity play an important role in solvation of the dye upon excitation, indicating that the nonspecific solvent effect is the factor contributing the most to the fluorescence spectral shifts. Moreover, a marked red shift is observed with increasing both the polarizability and the dipolarity of the environment for the compound (negative value for *a* and *b*), whereas an increase in acidity and basicity of the medium causes a weak blue shift in the fluorescence maximum (positive value for *c* and *d*). However, the latter terms are negligible in fluorescence analysis since they are characterized by the lowest values and high standard errors. Thus, the overall shift of the fluorescence maximum is bathochromic in more polar solvents due to a high contribution of SdP and SP in the regression. This means that the excited state’s energy level decreases via these parameters, and evidently, the stabilization of the excited state increases by increasing solvent polarity.

Since the shift of fluorescence maximum with an increasing of the solvent polarity is higher than the absorption shift, it may be concluded that there is better stabilization of the excited electronic state relative to the ground state in a more polar environment, and there is a rise in the dipole moment upon excitation. Analysis of the contribution percentages of different polarity parameters highlights the importance of the solvent dipolarity that is responsible for the Stokes shift, whereas the solvent polarizability, acidity, and basicity have a moderate influence. However, the correlation results, obtained according to eq 15 (Table 2), imply that the solvent polarizability is subjected to a large error and is negligible. Furthermore, the acidity and basicity terms, characterized by the lowest values and significant standard errors in the Stokes shift analysis, diminished the influence of these parameters. A high contribution of the solvent dipolar effect may result from a balanced contribution of the two opposing effects, that is, two aryl moieties constituting π-electronic entities and an oxazolone ring acting as an electron-accepting group, that cause a separation of charges by the creation of dipolar structures oriented differently in space. Such behavior was observed in compounds with a substituent displaying a low or moderate effect.^95^

Negative values of the coefficients *a*, *c*, and *d* indicate a decrease in the shift between absorption and fluorescence spectra with an increasing polarizability SP, acidity SA, and basicity SB, while the increase of the Stokes shift of the dye in the Catalán solvent scale results in solvent dipolarity (positive value for *b* coefficient). Following the experimental data, the changes in the Stokes shifts with solvent type are rather small, that is, they vary from 4200 cm^–1^ in MCH to 5261 cm^–1^ in MeCN and 5107 cm^–1^ in MeOH. The values of the Stokes shift are indicative of the charge transfer transition differences in the shift when changing the solvent from nonpolar to highly polar.

Analysis of the solvent dependence of the FQY for the Ox-π,π-Ph chromophore indicates that the dipolarity and basicity of the solvent contribute to the decrease in the FQY (negative sign of the parameters), whereas the polarizability and acidity of the solvent had a positive impact on the FQY. However, the low contribution percentages of acidity and basicity terms as well as high standard errors for the estimated coefficients diminished the influence of these parameters on Ox-π,π-Ph FQY.

### Linear and Nonlinear Spectroscopic Properties: Theoretical Aspects

To characterize the nature of the electronic transitions and to assign the bands observed in the experimental absorption spectra, the theoretical spectral properties were determined. The obtained excitation energies (λAb) are given in Table 3.

**Table 3:** Theoretical Linear and Nonlinear Spectroscopic Properties of the Ox-π,π-Ph Isomers

	GP		water	
	*Z*	*E*	*Z*	*E*
λ_Ab_^vert^ (nm)	406.97	422.37	422.61	444.80
λ_Ab_^cLR^ (nm)	411.36	423.85	415.79	425.79
λ_FL_^vert^ (nm)	491.63	487.08	529.68	475.15
μGS (D)	1.83	1.55	2.70	2.25
*x*	1.16	0.16	1.51	0.39
*y*	0.10	–1.54	–0.15	–2.22
*z*	–1.41	0.00	–2.23	–0.00
μCT (D)	3.98	3.10	5.40	5.74
*x*	–3.79	–3.24	–3.84	5.21
*y*	2.67	1.18	4.08	0.96
*z*	0.30	0.00	–0.65	0.00
linear polarizabilities				
*xx*	359.84	582.97	496.97	801.05
*xy*	19.45	–6.98	32.14	–15.11
*yy*	218.42	215.37	318.44	317.37
*yz*	23.94	0.00	41.19	0.00
*zx*	–20.48	0.00	–29.38	0.01
*zz*	172.29	110.39	247.01	153.94
⟨α⟩ (a.u.)	250.18	302.91	354.14	423.94
Δα (a.u.)	171.23	429.84	226.99	582.94
first-order hyperpolarizabilities				
*xxx*	–534.93	2396.07	–568.88	7889.80
*xyy*	–153.95	31.70	–443.31	87.80
*xyz*	–2.03	0.00	–7.36	–0.03
*yzz*	–22.96	–18.38	–51.80	–60.45
*yxx*	541.06	189.09	1656.25	647.33
*yyy*	227.01	35.90	683.51	55.87
*yzz*	0.18	13.49	37.48	34.25
*zxx*	–138.32	0.00	–232.65	0.67
*zyy*	–6.15	0.00	–53.42	0.09
*zzz*	60.37	0.00	71.96	–0.03
β_vec_ (a.u.)	206.66	10.05	332.99	278.98

The effect of the solvent on λAb is shown in Table S8. As previously reported,^29,92,96,97^ calculations of the linear optical properties were performed using only PBE0. Considering the vertical values, the values closest to the experimental ones are obtained for the *E* isomers. The average error for these conformers is 19.02 nm. For *Z* isomers, the maximum λAb shifts toward longer waves, and the relative error of the measured values increases to 29.93. Employing the state-specific corrected linear response (λ_Ab_^cLR^) approach increases the size of this displacement (Table S9). Taking into account λ_Ab_^vert^ and λ_Ab_^cLR^, it should be assumed that the occurrence of the tested dye in the form of a *Z* isomer will cause a bathochromic shift relative to *E* by an average of 25 nm. Moreover, the position of the absorption maximum band of *E* and *Z* isomers is sensitive to changes in the environmental polarity. Increasing the solvent polarity results in a bathochromic shift of the λ_Ab_^vert^ and λ_Ab_^cLR^. However, the nonmonotonous behavior of λAb is observed. The most intense maximum above 450 nm is almost a pure HOMO → LUMO transition for each isomer. However, the contributions from other orbitals are not negligible. Therefore, an additional maximum shifting toward shorter wavelengths may appear. In the case of the *Z* isomer, it is associated with HOMO–1 → LUMO and HOMO–2 → LUMO transitions, while for the *E* isomer, it is associated with HOMO–1 → LUMO and HOMO–4 → LUMO transitions.

The use of PBE0 also leads to reliable maximum fluorescence (λFL) results (Table S10). Similar to λAb, the closest measured values are obtained for the *E* isomer, with an average error of 2.89 nm. Isomerization causes a significant bathochromic shift, and the error increases to 58.84 nm for *Z*. In this case, the maximum λFL shift cannot be clearly determined during *E–Z* isomerization, as this relationship is closely dependent on the solvent. For example, Δλ_FL_^*E*–*Z*^ in Bu_2_O is 4.84 nm and in DMSO is 71.1 nm. In addition, in contrast to the experimental values, theoretical ones exhibit nonmonotonous behavior in the function of solvent polarity. In conjunction with absorption considerations, therefore, specific interactions in the solvent–solute system are to be expected, as well as the occurrence of H-bonds. This is confirmed by microsolvation studies performed for the *trans* isomer (Figure S9), taking into account two solvent molecules in the environment of the studied molecule. In the case of solvents for which an increase in excitation energy is observed during transition to a more polar solution (MeCN, H_2_O), a hydrogen bond is formed in the solvent–solute system.

The dipole moment values for both conformers of the Ox-π,π-Ph chromophore are presented in Figure 4 (graphs a and b) and Table S11. First, for both isomers μ_GS_ and μ_CT_, there is an increase as a function of solvent polarity. However, the Δμ_CT–GS_ does not exceed 3.9 D for both isomers in the tested solvents. The highest values of μ_GS_ were observed in water for both isomers, whereas the calculated values of the dipole moments for the CT excited state were at a maximum in THF (μ_CT_ = 5.53 D) and in Et_2_O (μ_CT_ = 5.84 D) for *cis* and *E* isomers, respectively. Moreover, the *Z* isomer reveals a slightly higher μ_GS_ relative to the *E* one irrespective of the solvent used (Δμ_GS_^*Z*–*E*^: 0.28 → 0.45 D from vacuum to water). Meanwhile, the *E* isomer is characterized by a higher value of the CT excited state dipole moment relative to *Z* isomer (Δμ_CT_^*E*–*Z*^: 0.88 → 0.34 D from vacuum to water).

The polarizability and hyperpolarizability of the molecule irradiated with an intense laser light is given when the electric field is the subject of much research in terms of understanding various linear and nonlinear optical properties (NLO). In particular, these studies include the inter-relationship of nonlinear optical properties with the electronic structure to design new multifunctional molecules. The calculated values for *E*/*Z* conformers are collected in Tables 3 and S12. For both *E* and *Z* conformers, the α, Δα, and β_vec_ values increase monotonously with the solvent polarity. For the *E* isomer, a higher value of α and a smaller β_vec_ relative to *Z* isomer are observed. Moreover, for both conformers, linear polarizability is higher than first-order hyperpolarizability.

### Optical Gain Investigations

**Figure 5:**
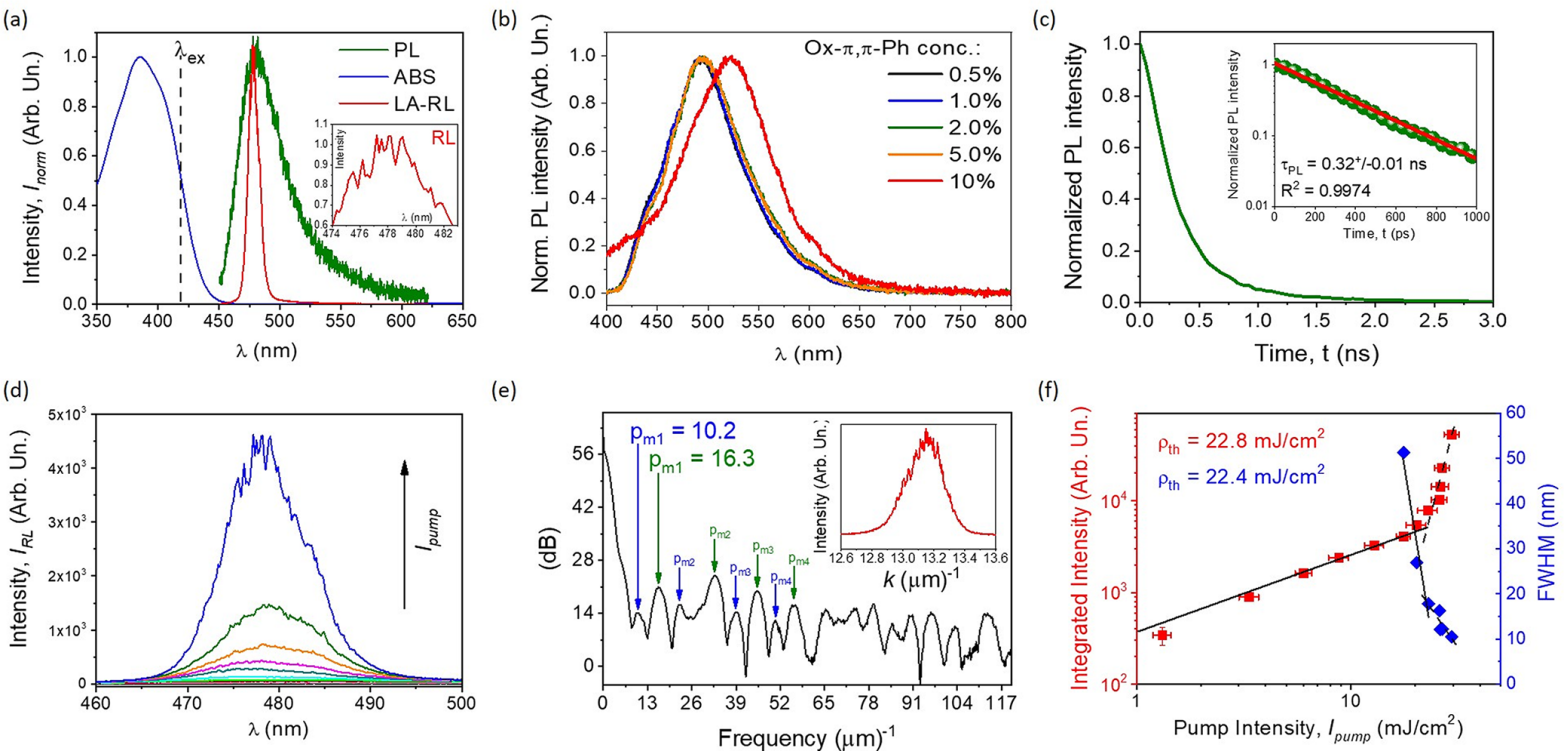
Absorption (solid blue), fluorescence (solid green), and light amplification in the way of random lasing (solid red) spectra of the Ox-π,π-Ph laser dye and its pump beam wavelength (dash black) (a); in the inset, enlarged RL sharp spikes are shown; PL spectra in dye concentration function (b); fluorescence lifetime decay curve, and in the inset, its enlarged fragment, in (*y*)log scale (c); LA spectra induced by increasing the pump beam intensity (d); PFT analysis defining the optical gain resonator size based on the RL spectrum (inset), where the *x* axis is presented as *k* (1/μm) (e); and the RL energy threshold estimated in two ways: spectra integration (red color) and fwhm method (blue color) (f).

The spectroscopic properties of the considered thin film was presented in Figure 5. The Ox-π,π-Ph multifunctional chromophore (2% dye content) absorbs the light in the UV–vis range of the spectrum, namely, 300–450 nm (with maximum localized at 385.2 nm; Figure 5a), whereas the fluorescence spectra were collected in the range of 450–550 nm (maximum positioned at 480.6 nm, full width at half-maximum is equal to 43.7 nm). However, the stimulated emission (ST) was also observed at 478.0 nm, and its fwhm parameter was estimated as 9.8 nm. The last-mentioned value indicates that the optical gain profile (significant spectral narrowing) was observed. The inset (Figure 5a) shows separated laser mode maxima, which indicates a random lasing character of the obtained emission. However, the PL spectra were also collected for the set of polymeric films with increasing laser dye concentration (Figure 5b). The dye content in the range of a 0.5–5.0% dry w/w ratio with polymer seems to characterize the same spectroscopic character, with a stable fluorescence at the same wavelength. However, the most concentrated sample gave a PL signal with the significant red-shifted maximum at 521.2 nm, which is probably implied from the *J*-aggregate formation. For further studies, only the sample containing 2% of the laser dye was taken into account. The fluorescence lifetime of the Ox-π,π-Ph chromophore was shown in Figure 5c, and its time constant of PL decay curve was estimated at 0.32^+^/–0.01 ns, with high precision of the implemented monoexponential function approximation (inset). The individual output spectra when the sample was induced by increasing the *I*_pump_ value are gathered in Figure 5d. Such a gain profile can be investigated using the PFT analysis, where the RL emission wavelength is transferred into the *k* wave vector expressed in μm^–1^ (Figure 5e). That approach leads to defining the frequently repeated maxima values, characterizing the collected emission, and defining the optical resonator dimension according to the well-described models. Two of them were considered in this Article, namely, WGM^98−100^ and Fabry–Perot.^101,102^ In Figure 5e, the less and more significant maxima marked in blue and green colors, accordingly, were taken under consideration. The estimated and averaged optical resonator sizes were gathered in Table 4. Approximated optical resonator sizes are in agreement with similar ones, for both models (WGM and Fabry–Perot) currently available in the literature (which are in the range of tens of microns).^98−102^ Finally, the energy threshold value to obtain an efficient light amplification process in the random lasing fashion was estimated according to the relation between the output integrated intensity or fwhm parameter and the pump beam intensity (Figure 5f). The ρ_th_ value was calculated as 22.8 and 22.4 mJ/cm^2^, respectively. The estimated values indicate that the system geometry might be optimized in order to reduce this parameter as it was shown in literature for the pyrazolines group of the laser dyes.^103^

**Table 4:** Optical Resonators Size Analysis

	*p*_m1_^a^	*p*_m2_^a^	*p*_m3_^a^	*p*_m4_^a^	*p̅*_m_^a^	*p*_m1_^b^	*p*_m2_^b^	*p*_m3_^b^	*p*_m4_^b^	*p̅*_m_^b^
WGM	13.4	14.9	17.3	16.8	15.6	21.7	21.8	20.1	18.6	20.5
Fabry–Perot	21.1	23.4	27.2	26.4	24.5	34.1	34.3	31.5	29.3	32.3

a The weaker signal series on the PFT chart (Figure 5c).

b The stronger signal series on the PFT chart (Figure 5c).

### All-Optical Switching Phenomenon

**Figure 6:**
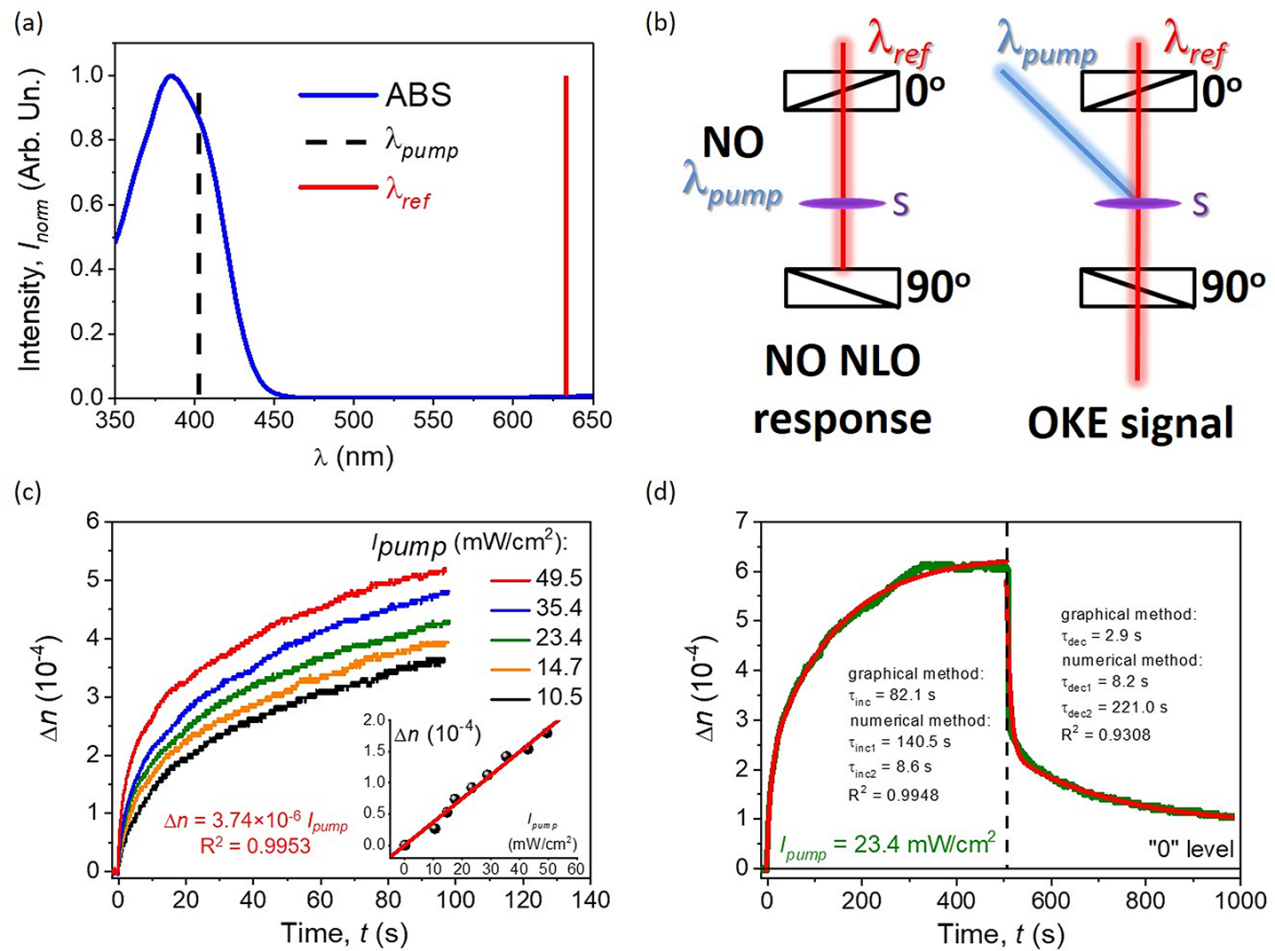
Absorption spectra of the Ox-π,π-Ph NLO chromophore together with marked λ_pump_ (405 nm) and λ_ref_ (638 nm) in the black dashed line and the solid red line, respectively (a); scheme of the pump–probe experimental setup, when the inducing beam is utilized (right panel) and when it is not utilized (left one) (b); Δ*n* kinetics induced by various *I*_pump_ values; as the inset in a linear correlation between photoinduced birefringence and *I*_pump_ (c); complete photoinduced birefringence kinetics (up to the plateau range), together with its thermodynamic decay curve (d). Full description in the text.

To follow the general requirements of the pump–probe laser setup, we have utilized two different wavelengths. The first one was inscribed into the absorption resonance of the active medium (marked in dashed black line in Figure 6a) and served as the pump beam (Figure 6b, right panel). On the other hand, the wavelength of the reference laser line marked in red in Figure 6a,b was out of the absorption range of the investigated organic system. In this way, the probe line monitors the refractive index conditions and all kinds of changes (thanks to the introduced cross-polarizer system), whereas the pump beam modulates optical parameters, including a phase change causing the refractive index indicatrix deformation and a second, nonlinear *n*2 generation (Figure 6b). When the inducing laser line is not applied, the output signal is not achieved as well. It is because of the presence of the initial optical isotropy that the organic system is characterized without any external stimuli. The kinetics of the photoinduced optical birefringence when the applied *I*_pump_ is increased is shown in Figure 6c. The inset presents a linear correlation between the applied laser light excitation intensity and the generated Δ*n*, which confirms the existence of the optical Kerr effect in the considered hybrid system.^60,61^ The maximum gained photoinduced birefringence in the Ox-π,π-Ph/PMMA system was 5.3 × 10^–4^, which was acquired after about 100 s of UV laser light treatment. Moreover, based on the above relation, the *n*2 was estimated and it is equal to 3.74 × 10^–6^ cm^2^/mW (2.89 × 10^–8^ m^2^/W), which is consistent with the similar organic systems characterizing the all-optical switching behavior recently shown in the literature.^22,29,104−107^ Namely, a pyrazolone derivative gave the maximum photoinduced birefringence equal to 5.5 × 10^–4^, whereas the *n*2 parameter was estimated at (4.1 ± 0.2) × 10^–6^ cm^2^/mW, which indicates that the NLO properties are on the same order of magnitude like the investigated molecular hybrid system.^29^ Then, comparing the current results with the other low-molecular NLO chromophores, such as thiophene or pyrazoline derivatives, their *n*2 parameter was estimated around 1.6 × 10^–7^^22^ and 3.9 × 10^–7^ cm^2^/mW,^104^ respectively, which are even 1 order of magnitude lower values. While making another comparison between the investigated organic system and the photochromic polymers,^105,106^ or polymers doped with photosensitive azo-functionalized polyhedral oligosilsesquioxane (POSS),^107^ the conclusions are the same. The experimental results given in the literature show that the multifunctional organic system introduced in here is highly effective due to the remote optical anisotropy modulation. Furthermore, it can even be competitive with much more expanded systems, like photochromic or functionalized polymers.

The so-called “static” (or total) photoinduced birefringence kinetics was analyzed in detail and graphically presented in Figure 6d. For both and increase and decrease in Δ*n*, a total of 500 s was devoted. A photostationary state (plateau range; Δ*n* saturation) was achieved after about 300 s (5 min), and during the next 200 s it was stable. Afterward, when the pump laser was in the “OFF” state (marked by black dashed line in Figure 6d), a significant signal drop was observed, and after about 500 s the Δ*n* value reached close to 1 × 10^–4^. Such an observed signal indicates the reversibility level (on the same time scale as signal recording) at about 83%. Photoinduced birefringence kinetics was analyzed in two various approaches, the graphical and analytical ones. The first one brought time constants for a signal increase and decrease as follows: τ_inc_ = 82.1 s and τ_dec_ = 2.9 s, respectively. These values define the photocontrolled process as the slower one, which needs a lot of energy to achieve efficient molecular reorientation. Indeed, the thermal dark relaxation process is energetically preferred and much faster (1 order of magnitude lower). If considering the numerical data analysis, the biexponential approximation function was the best solution (*R*^2^ was equal to 0.9948 and 0.9308, accordingly). In this case, two time constants were found for each process. τ_inc1_ = 140.5 s and τ_inc2_ = 8.6 s, while the reverse spontaneous process is described by the following numbers: τ_dec1_ = 8.2 s and τ_dec2_ = 22.1 s, respectively. For the photoinduced Δ*n* state, the first step seems to be much slower, which needs a lot of energy to reorient the active molecules. The second part, when a majority of available fragments are aligned, it is faster and leads to the photostationary state. However, the reversible process first is faster (significant signal drop) and then much slower, which demands much more time to provide the initial molecular misalignment. Indeed, after the first 10 s of thermal darkness conditions, about 40% was obtained before Δ*n* is reduced (Figure 6d). Estimated time constant values in both approaches are in a good agreement with their equivalents for previously cited low-molecular NLO active systems, as well as the photochromic polymers.^22,29,104−107^ Finally, the third-order susceptibility parameter was estimated at 2.3 × 10^–10^ (m/V)^2^. Comparing this parameter with the one quoted earlier, it can be stated that the fourth-order NLO tensor is similar to the thiophenes (χ^(3)^ = 2.4 × 10^–10^ (m/V)^2^) or pyrazolines (χ^(3)^ = 1.8 × 10^–11^ (m/V)^2^), respectively.^22,104^ whereas in the pyrazolone derivative system, it was observed to be a 3 orders of magnitude higher third-order NLO susceptibility (χ^(3)^ = 1.1 × 10^–7^ (m/V)^2^)^29^ than in the case of the considered organic system.

**Figure 7:**
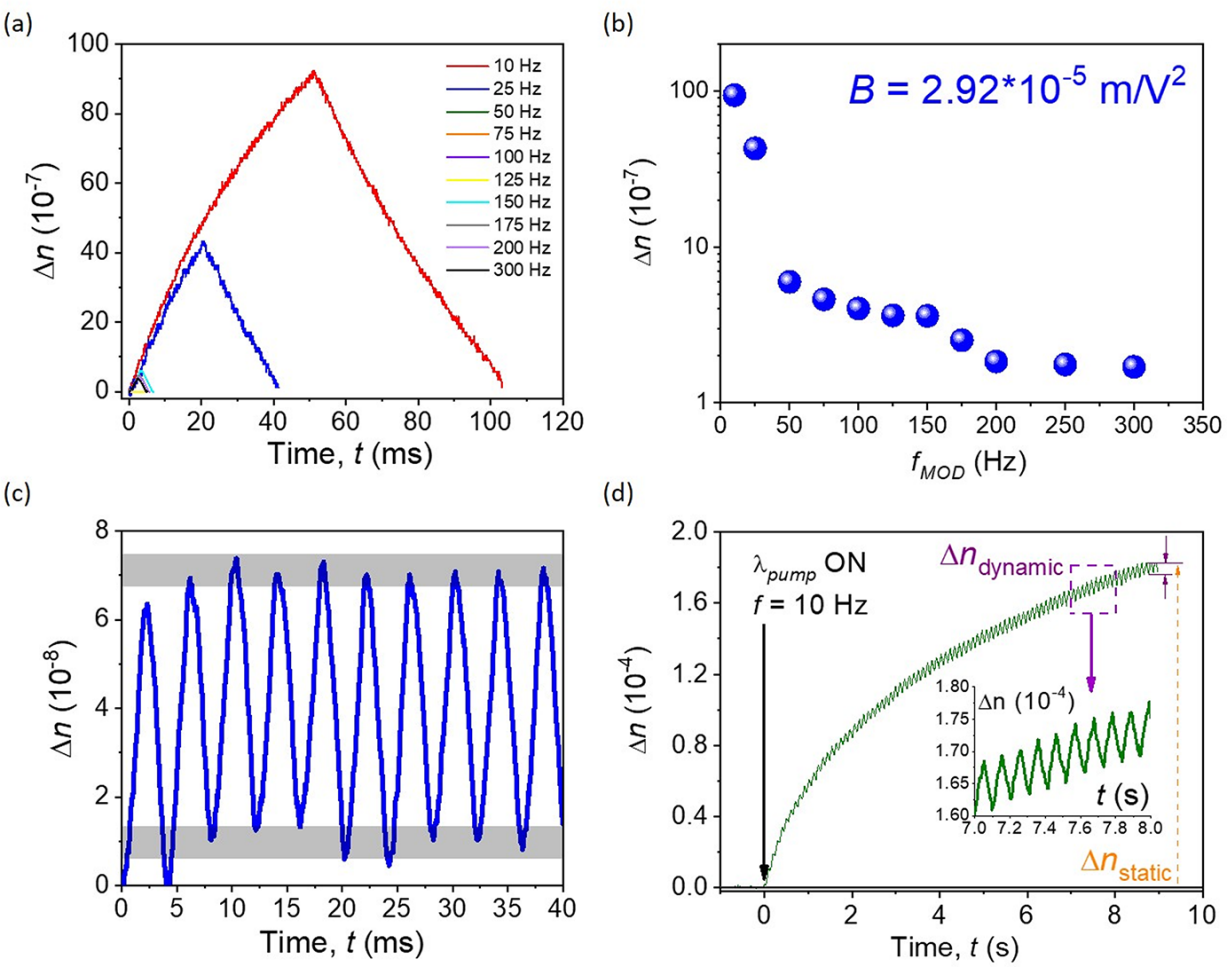
Kinetics of the dynamic part of Δ*n* (*trans–cis–trans* conformational changes), when various signal modulations are applied (*f* in the range 10–300 Hz) (a); photoinduced birefringence amplitude of the dynamic OKE signal (b); multiple NLO dynamic responses for the implemented modulation frequency 200 Hz (c); *I*_pump_ for (a)–(c) = 22.2 mW/cm^2^; and both static and dynamic Δ*n* inducement when *I*_pump_ is equal to 23.4 mW/cm^2^ and modulation frequency is applied (*f* = 10 Hz) (d); inset shows enlarged dynamic OKE signal modulation.

As a supplement to the static photoinduced birefringence analysis, the dynamic ones (Δ*n*_dyn_) were provided in Figure 7. Light driven molecular transformations (*E-Z-E* or, in particular, *trans–cis–trans*) can also be determined using the OKE experimental setup. Since the active medium is modulated by the pump laser channel with various lightning frequencies, the efficiency of the aforementioned transitions can be observed. The highest molecular transformation was observed for the lowest modulation frequency, 10 Hz (Figure 7a,b). However, such a signal modulation can be observed also under higher *f* values, up to the 300 Hz (or higher), and what is more important, the Δ*n*_dyn_ changes are still significant (Figure 7b). Furthermore, based on the experimental results, the Kerr constant (*B*) was estimated to be equal to around 2.92 × 10^–5^ m/V^2^. Such a number is slightly higher than the other numbers, defined for various low-molecular NLO active systems, like the thiophene derivative (*B* = 3.6 × 10^–6^ m/V^2^)^22^ or pyrazolines (*B* = 4.1 × 10^–7^ m/V^2^ for DCNP,^104^ or *B* = 3.9 × 10^–7^ m/V^2^ for PY-*p*NO_2_^108^ molecules), or significantly lower than for the pyrazolone derivative (3.5 × 10^–3^ m/V^2^).^29^ Additionally, the multiple NLO dynamic responses for the implemented modulation frequency at 200 Hz was acquired, and it is presented in Figure 7c. Gray panels indicate the minima and maxima of the collected signals localized almost on the same Δ*n*_dyn_ level and prove their high stability over time and pump line modulation. Interestingly, the dynamic component of the photoinduced birefringence is clearly visible when *I*_pump_ is modulated (*f* = 10 Hz) and the time scale is elongated to the range of seconds (typically used for the static OKE measurements). It confirms the mechanism of the observed third-order NLO phenomenon.^60,61^

### Second and Third Harmonic Generation

**Figure 8:**
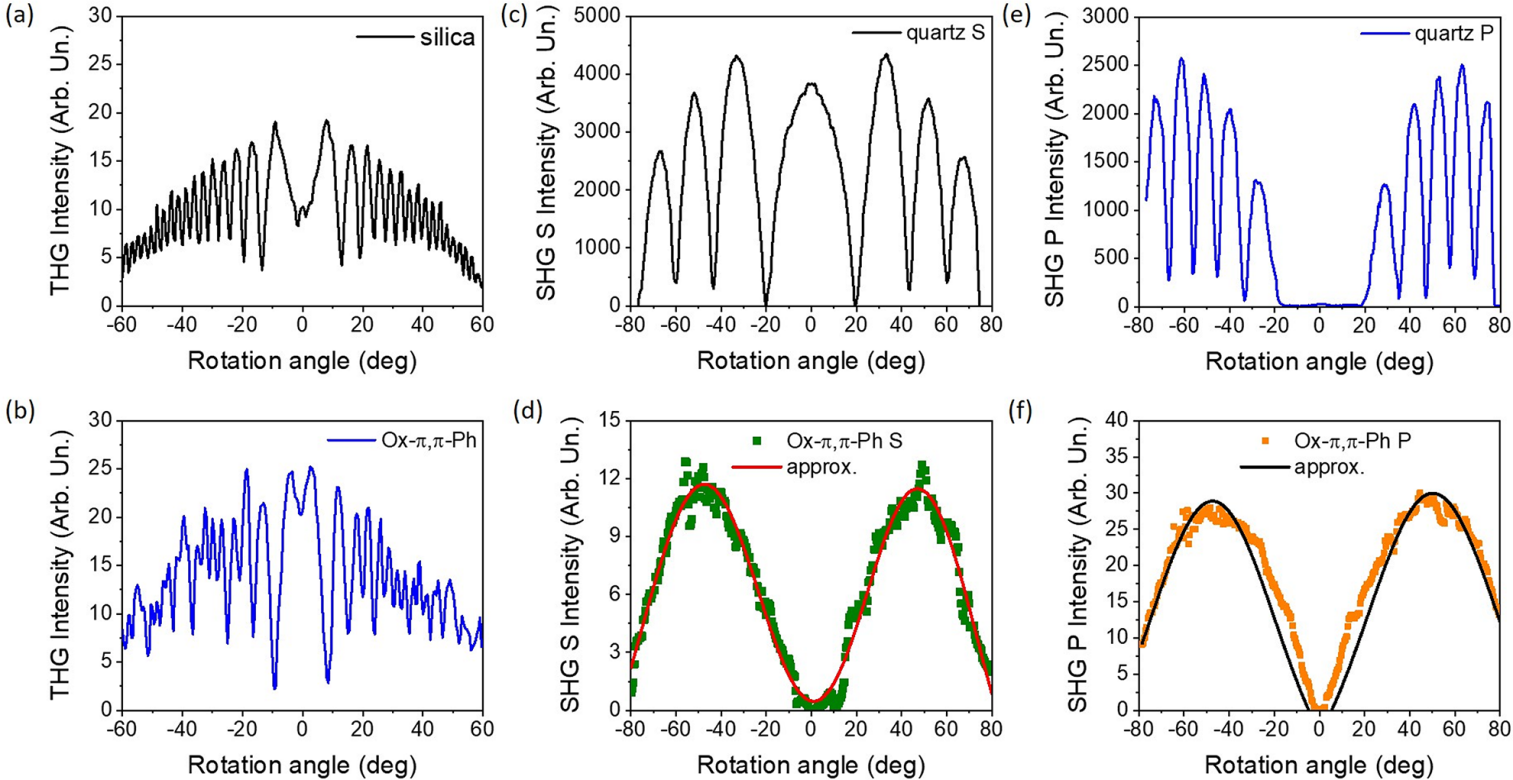
THG signal in the shape of Maker fringes for the reference material (silica) (a) and investigated NLO chromophore (b); SHG signal of the reference material (quartz) in *S* and *P* polarization configurations (c, e) and the same results for the Ox-π,π-Ph NLO chromophore, respectively (d, f).

The NLO response of the investigated organic system in the context of higher harmonics of light generation is presented in Figure 8. If considering the THG signal (Figure 8a,b), and comparing with the reference material, the Ox-π,π-Ph NLO chromophore characterizes more efficient electron cloud distribution modulation under a fundamental laser beam, which implies it has a three times higher third-order NLO susceptibility (χ^(3)^ = 6.0 × 10–22 m^2^/V^2^). Such a property could be significantly improved by minimizing the sample thickness below 1 μm. The optical path determines any kind of absorption, but also the reabsorption processes, which in fact were taken under consideration during the χ^(3)^ value estimation. However, since there is less external stimuli or signal aberration and, that is, the reabsorption processes can be neglected, then the output feature is more reliable due to its character and value. Whereas the frequency-generated wavelength is doubled, it is less efficient in both experimental setup polarization configurations. Indeed, the χ^(2)^ parameter of the organic active system is equal to 0.05 and 0.14 pm/V for *S* and *P* laser light polarization directions, respectively. Since the quartz characterizes the same tensor value as 1.00 pm/V, the investigated organic system should not be considered as the efficient SHG signal generator. It is important to note that the reabsorption processes were also taken under consideration during the NLO parameters value estimation. Another reason for such an inefficient SHG signal from the organic system can be its thickness and applied external optical anisotropy. Since the corona poling was introduced, the polar molecule ordering depends on its efficiency, which is difficult to control. Additionally, the NLO chromophore concentration can strongly influence the SHG/THG signal magnitude, which was proven for a *push–pull* type group of pyrazoline derivatives, where a nonlinear optical response in dye content variable was investigated.^65^ For the numbers higher than 2% of NLO chromophore concentration, a THG output increase was noticed. Albeit, the idea of the performed investigation was to use exactly the same sample shape for all conducted experiments.

## Conclusions

In summary, we have introduced a newly synthesized laser dye, being an appealing alternative working as a multitask organic-based hybrid material. We have experimentally proven that the oxazolone derivative presents significant and remarkable spectroscopic features, which might be utilized in photonics, spectroscopy, and optoelectronics. It can be easily designed for light amplifier or light modulator construction, where full and remote control is expected. Furthermore, the observed light amplification phenomena can be guided by setup geometry, as the result might deliver particular laser modes. On top of that, the well-working all-optical switching process in the considered hybrid system at the same time may change its optical anisotropy and give the 0–1 optical output channel, which is necessary in many types of optoelectronic networks or computers. The multifunctional organic hybrid system reported here can be adopted in real-time holographic applications^109−111^ as an efficient light-sensitive remote-controlled medium. Such a created, self-organized disordered system constitutes a promising material for the fast manufacturing of organic and miniaturized lasers, which additionally can generate the higher harmonics of light as well. Aforementioned application versatility makes the oxazolone derivative a highly attractive organic laser dye/NLO chromophore for multiple employments. This family of compounds should undoubtedly be explored in the future in the context of other possible subjects, such as waveguiding.

## Supporting Information Available

NMR spectra of compounds, data from the spectroscopic measurements, and results from electronic structure calculations (PDF)
