# Supplementary material for: Multifunctional Oxazolone Derivative as an Optical Amplifier, Generator, and Modulator
Source: J Phys Chem B. 2022 Feb 18;126(8):1742–57. doi: 10.1021/acs.jpcb.1c08056 (PMC8900139; doi:10.1021/acs.jpcb.1c08056)
Supplement: Supplementary file 1 — jp1c08056_si_001.pdf [file jp1c08056_si_001.pdf]

Multifunctional Oxazolone Derivative as an Optical Amplifier, Generator and Modulator

ELECTRONIC SUPPLEMENTARY INFORMATION

Adam Szukalski,[*,†] *Przemysław Krawczyk,*[‡] *Bouchta Sahraoui,*[§] *Beata Jędrzejewska*[*,§]

[†]Wroclaw University of Science and Technology, Faculty of Chemistry, Wyb. Wyspiańskiego 27, 50-370 Wrocław, Poland; *e-mail: adam.szukalski@pwr.edu.pl
[‡]Nicolaus Copernicus University, Collegium Medicum, Faculty of Pharmacy, Kurpińskiego 5, 85-950 Bydgoszcz, Poland
[$]Laboratoire MOLTECH-Anjou, Université d'Angers, UFR Sciences, UMR 6200, CNRS, 2 Bd. Lavoisier, 49045, Angers Cedex, France
[§]Bydgoszcz University of Science and Technology, Faculty of Chemical Technology and Engineering, Seminaryjna 3, 85-326 Bydgoszcz, Poland; *e-mail: beata@pbs.edu.pl

Table of contents	Page
Synthesis method of Ox-π,π-Ph	S2
^{1}H NMR spectra	S3-S4
^{13}C NMR spectra	S5-S6
HMBC_^{15}N NMR spectra	S7
IR spectra	S8
Light amplification dedicated setup and other details – Figure S1	S9
Atom numbering scheme – Figure S2	S9
Results of a Potential Energy Surface (PES) scan for dihedral angle C6=C8-C9=C10 (wB97xd/6-33++G(d,p)) – Figure S3	S10
Time-dependent changes in absorption spectra of Ox-π,π-Ph in EtOAc after irradiation – Figure S4	S11
The normalized electronic absorption and fluorescence spectra of Ox-π,π-Ph in solvents of different polarities – Figure S5	S12
Normalized fluorescence excitation and fluorescence spectra of Ox-π,π-Ph in EtOAc recorded for different observation and emission wavelengths – Figure S6	S13
Normalized fluorescence excitation and fluorescence spectra of Ox-π,π-Ph in EtOAc recorded for solution of different concentration – Figure S7	S14
Comparison of the theoretical absorption maxima bands determined for monomer and dimer in different solvents – Figure S8	S15
Solvent parameters and values of solvent polarity functions – Table S1	S16
Structural parameters of the *E* isomer in the ground state – Table S2	S17
Structural parameters of the *Z* isomer in the ground state – Table S3	S18
Density difference plots – Table S4	S19-S20
The frontier orbital energies in selected solvents – Table S5	S21
CT parameters for the bright low-lying excited state of Ox-π,π-Ph – Table S6	S22
Solvatochromic spectral parameters of Ox-π,π-Ph. The corresponding coefficients were calculated using multivariable linear regression applying *Catalán* approach – Table S7	S22
The vertical excitation energies – Table S8	S23
The cLR corrected excitation energies – Table S9	S23
The vertical de-excitation energies – Table S10	S24
Interaction in the solvent-solute system in selected environments - Figure S9	S25
Calculated values of dipole moments for the ground and CT excited state – Table S11	S26
Nonlinear properties of Ox-π,π-Ph isomers – Table S12	S26

The synthesis method of 4-(3'-phenyl-2'-propenylidene)-phenylooxazol-5(4*H*)-one (its acronym Ox-π,π-Ph).

Hippuric acid (0.5 g, 2.8 mmol) in acetic anhydride (1.3 mL, 1.42 g, 15 mmol) was heated at 85°C under stirring until it became an intensely yellow color. Then, the cinnamaldehyde (0.35 mL, 0.37 g, 2.8 mmol) and sodium acetate (0.23 g, 2.8 mmol) was added and the solution was refluxed for 4 hours. After cooling it to room temperature, anhydrous ethyl alcohol (5 mL) was added and the mixture was refrigerated overnight. The crude product was purified by column chromatography (silica gel, TCM), affording a yellow solid (0.40 g). Yield 55%. Mp: 173−174°C. ^{1}H NMR (400 MHz, from TMS, $CDCl_3$) δ (ppm): 8.15-8.11 and 8.12-8.08 (d,d, *J*=16.0 Hz, 1H, =CH–), 8.09-8.07 (d, *J*=8.0 Hz, 2H, Ph), 7.60 (m, 3H, Ph), 7.57 (t, 2H, Ph), 7.41-7.39 (d, *J*=8.0 Hz, 2H, Ph), 7.38 (t, 1H, Ph), 7.31-7.28 (d, *J*=12.0 Hz, 1H, =CH–), 7.12-7.08 (d, *J*=16.0 Hz, 1H). ^{1}H NMR (400 MHz, from TMS, DMSO-d_6) δ (ppm): 8.03-8.01 (d, *J*=8.0 Hz, 2H, Ph), 8.02-7.98 and 7.99-7.95 (d,d, *J*=16.0 Hz, 1H, =CH–), 7.69 (t, 1H, Ph), 7.63 (t, 4H, Ph), 7.53-7.50 (d, *J*=12 Hz, 1H, =CH–), 7.47-7.45 (d, *J*=8 Hz, 2H, Ph), 7.43 (t, 1H, Ph), 7.42-7.38 (d, *J*=16.0 Hz, 1H, =CH–). ^{13}C NMR (100 MHz, form TMS, DMSO-d_6) δ (ppm): 123.2, 127.9, 128.1, 129.7, 129.8, 130.6, 133.7, 137.7, 145.4 for *C*H; 125.7, 133.8, 136.2, 161.4, 165.4 for *C*. IR (KBr, cm^{-1}): 681, 693, 748, 773, 883, 974, 1001, 1071, 1111, 1172, 1277, 1328, 1362, 1450, 1493, 1544, 1580, 1638, 1750, 1788, 2925, 3053, 3327.

^{1}H and ^{13}C NMR chemical shift data are in agreement with the dye structure. The ^{1}H NMR spectra show doublet at 7.5 ppm ascribable to the methine proton in a *Z* conformation ($^3J_{H,H}$ = 12 Hz) as well as two doublets (one with splitting into doublet-doublet) related to methine protons in a *E* conformation ($^3J_{H,H}$ =16 Hz) which are localized roughly at 7.4 and 8 ppm. Furthermore, the formation of oxazolone backbone was confirmed by strong bands observed in the IR spectra at ca. 1650–1780 cm^{-1}, 1500–1650 cm^{-1} and 1500–1610 cm^{-1} due to the C=O, C=N and C=C double bonds, respectively.

^{1}H NMR spectrum of Ox-π,π-Ph in $CDCl_3$

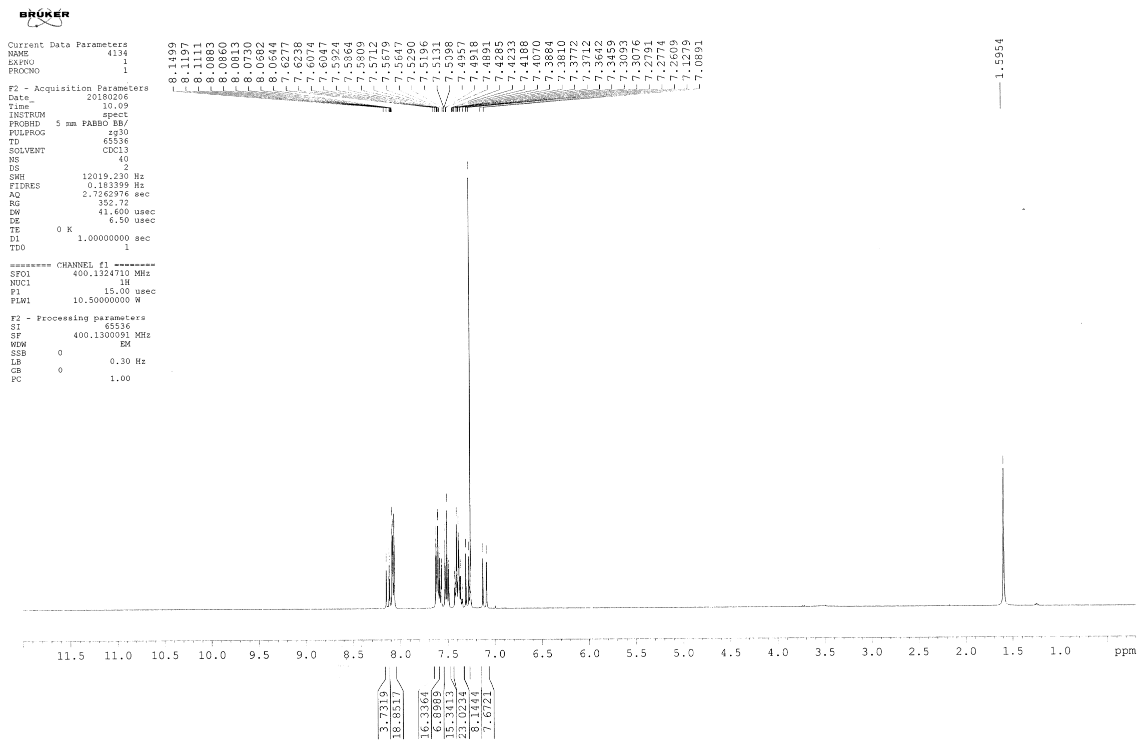

Enlarged spectrum in the range of 6-9 ppm.

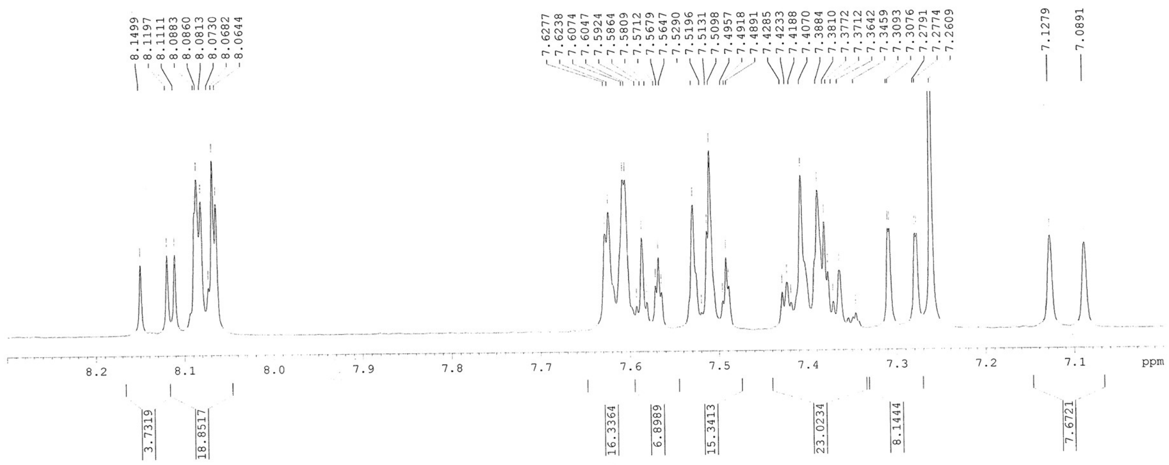

^{1}H NMR spectrum of Ox-π,π-Ph in DMSO-d_6

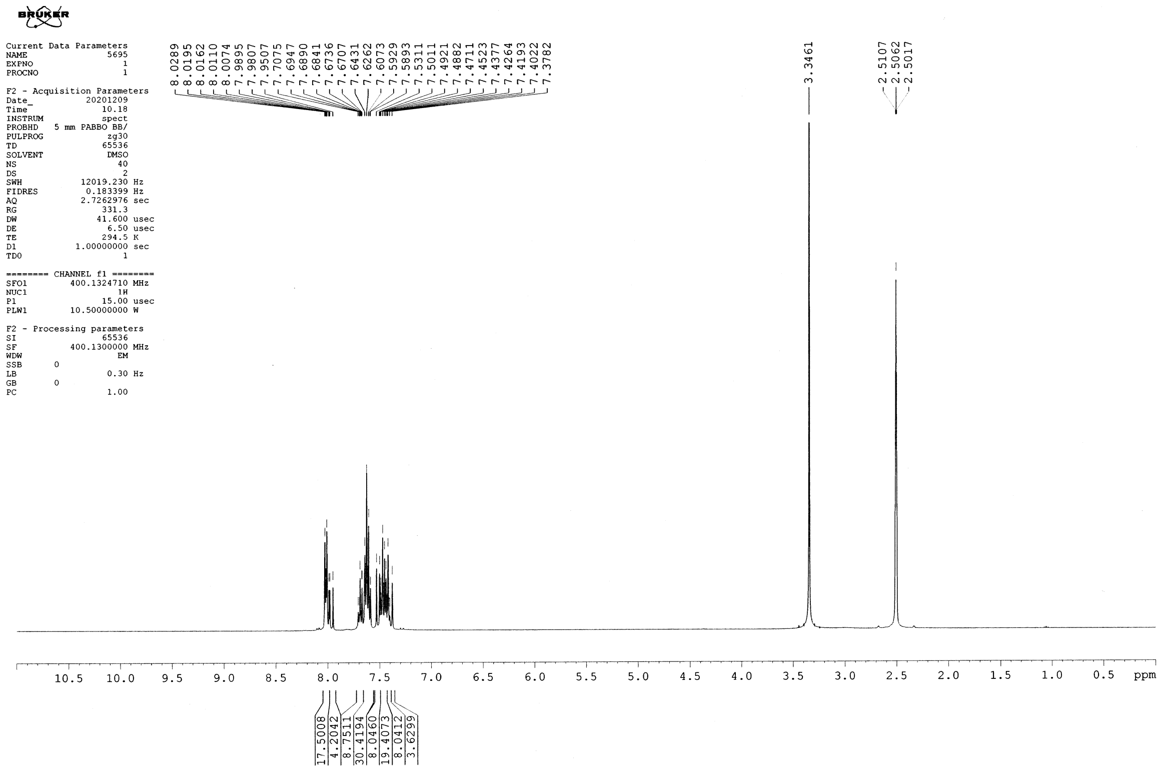

Enlarged spectrum in the range of 6-9 ppm.

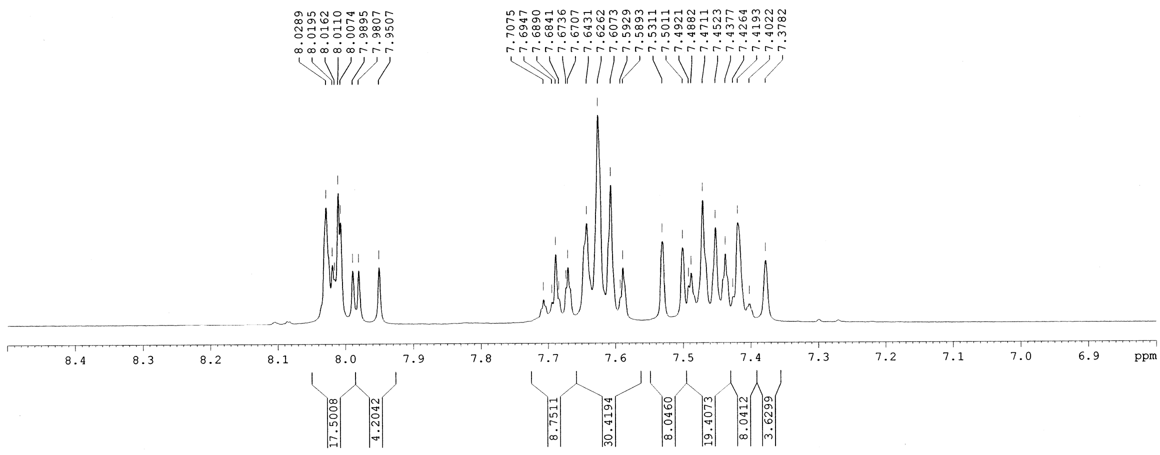

^{13}C NMR spectrum of Ox-π,π-Ph in DMSO-d_6

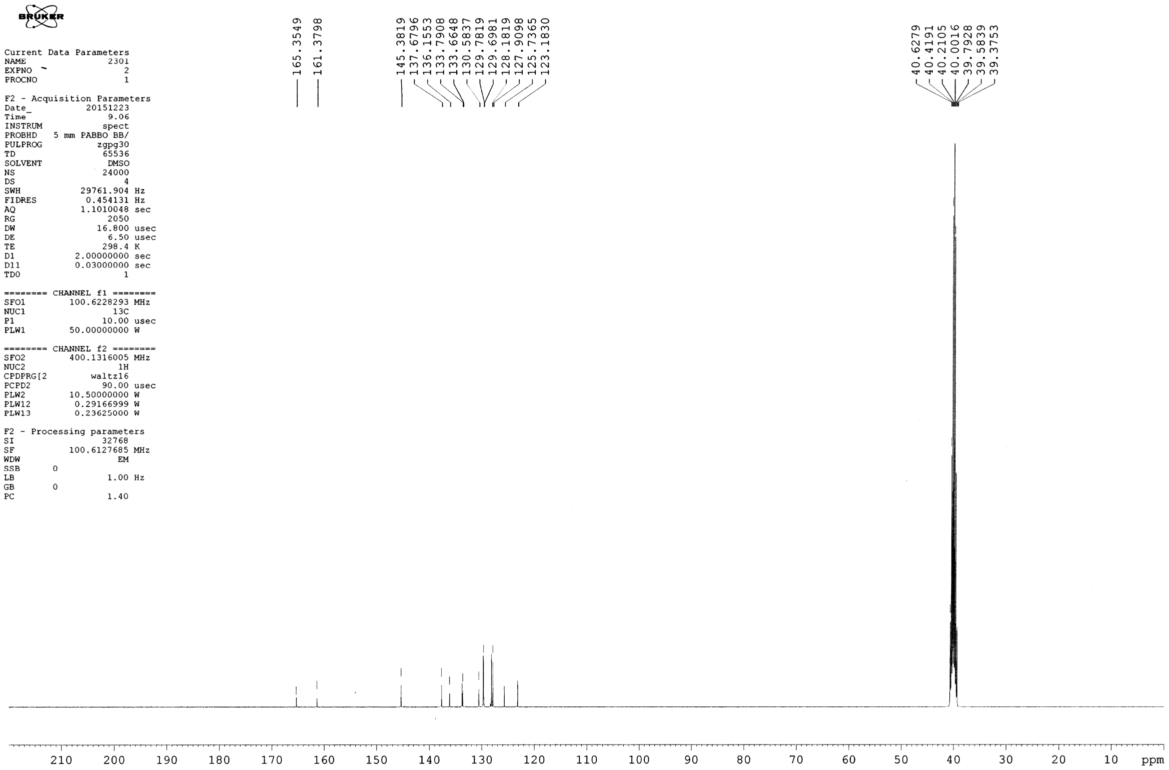

Enlarged spectrum in the range of 100-180 ppm.

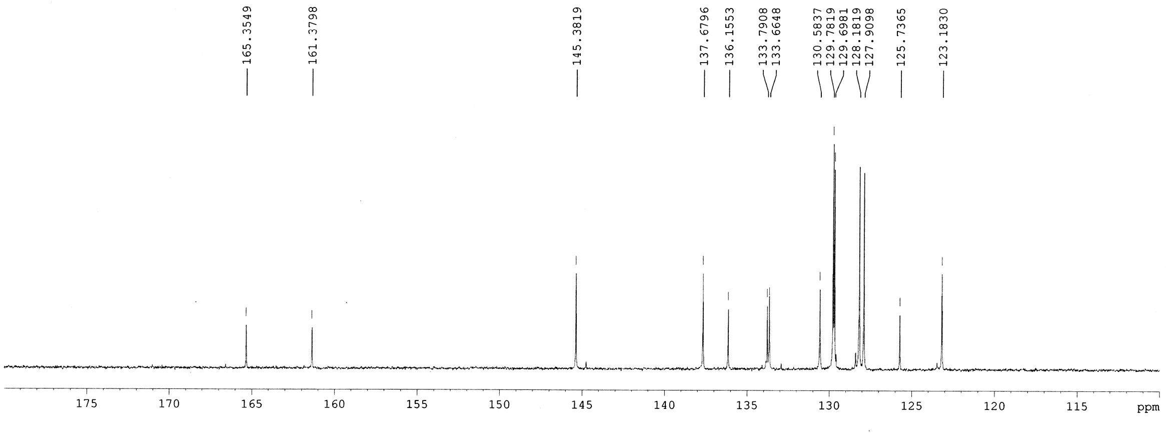

^{13}C NMR spectrum of Ox-π,π-Ph dept in DMSO-d_6

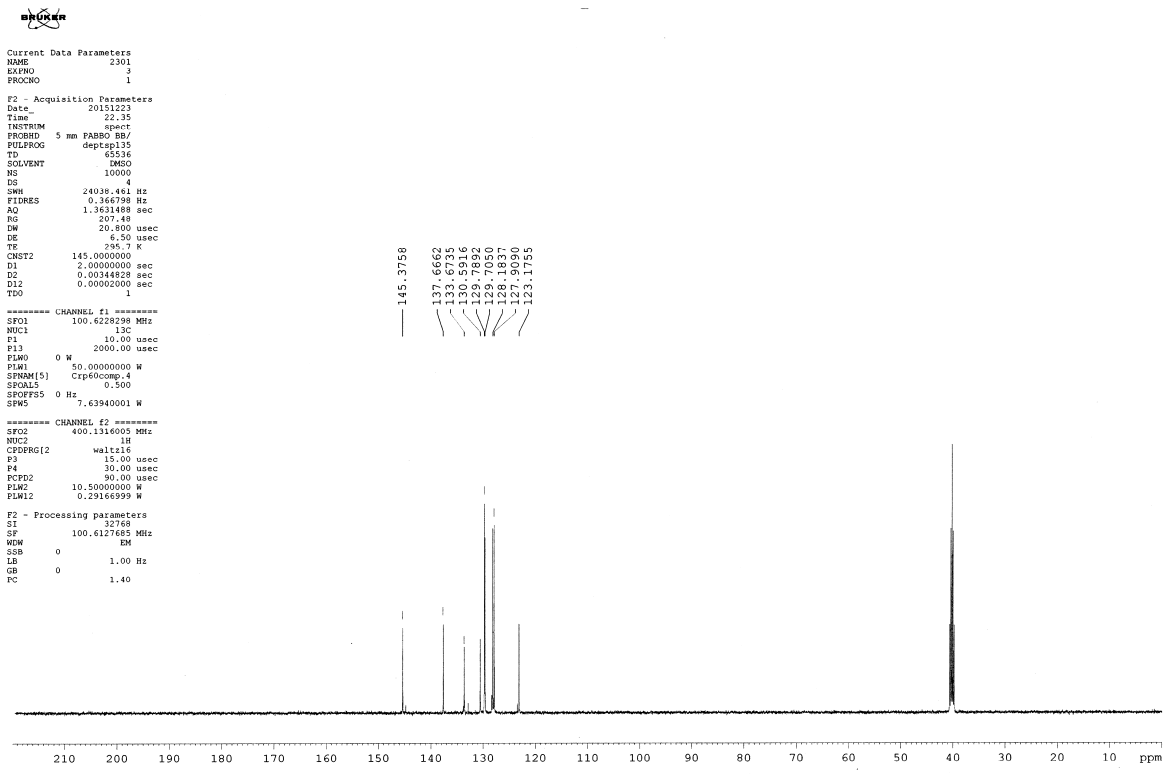

Enlarged spectrum in the range of 100-170 ppm.

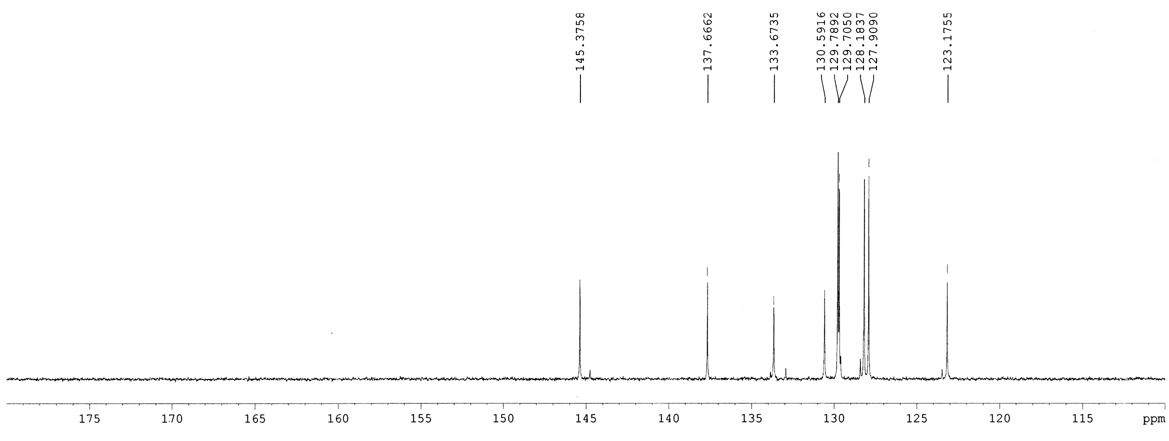

^{1}H–^{15}N HMBC spectrum of Ox-π,π-Ph in DMSO-d_6

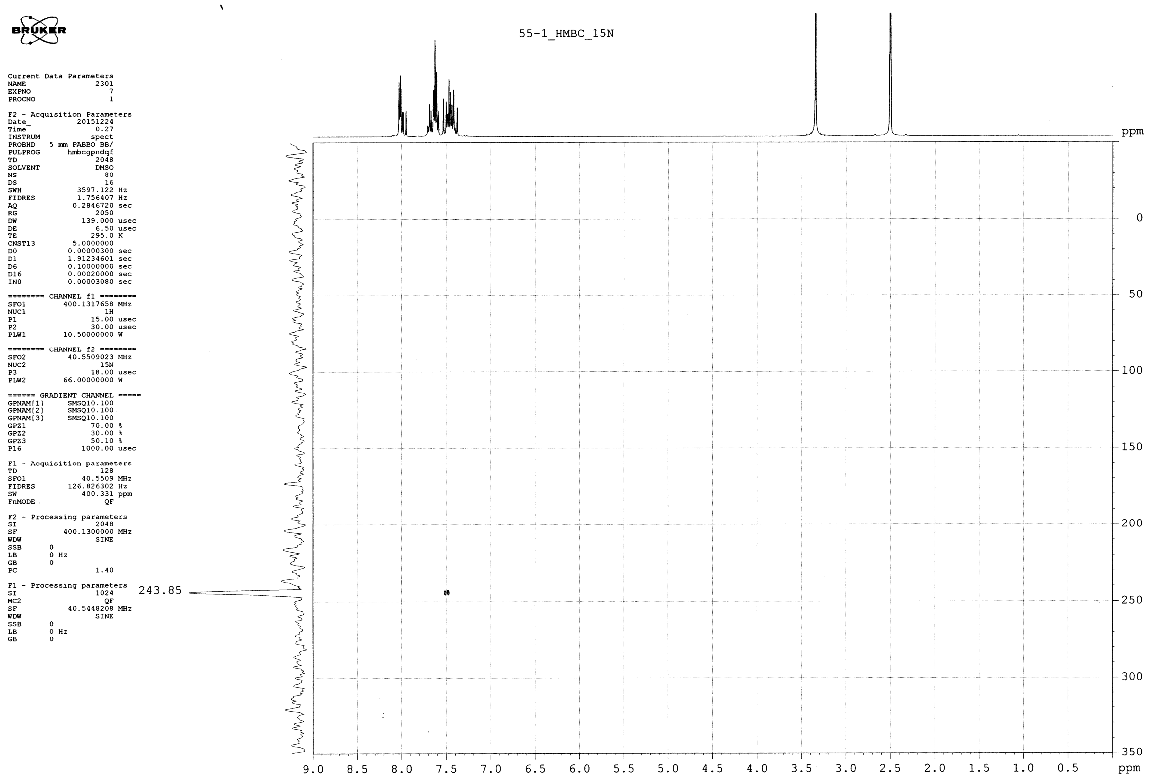

Enlarged spectrum

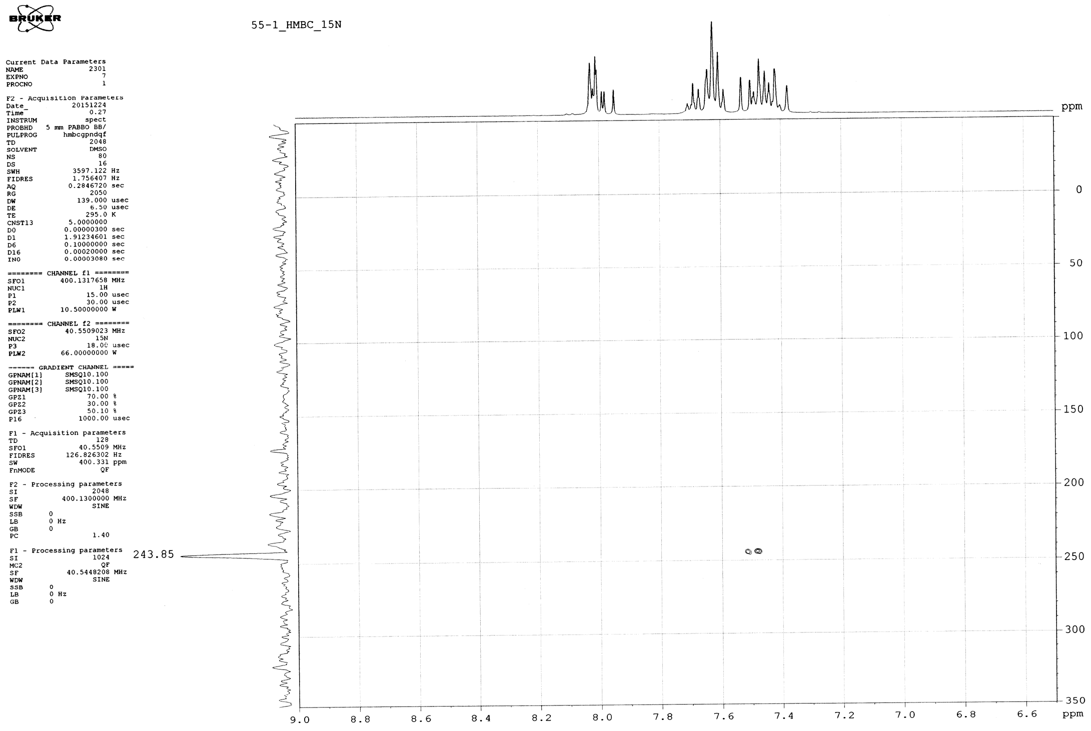

IR spectrum of Ox-π,π-Ph

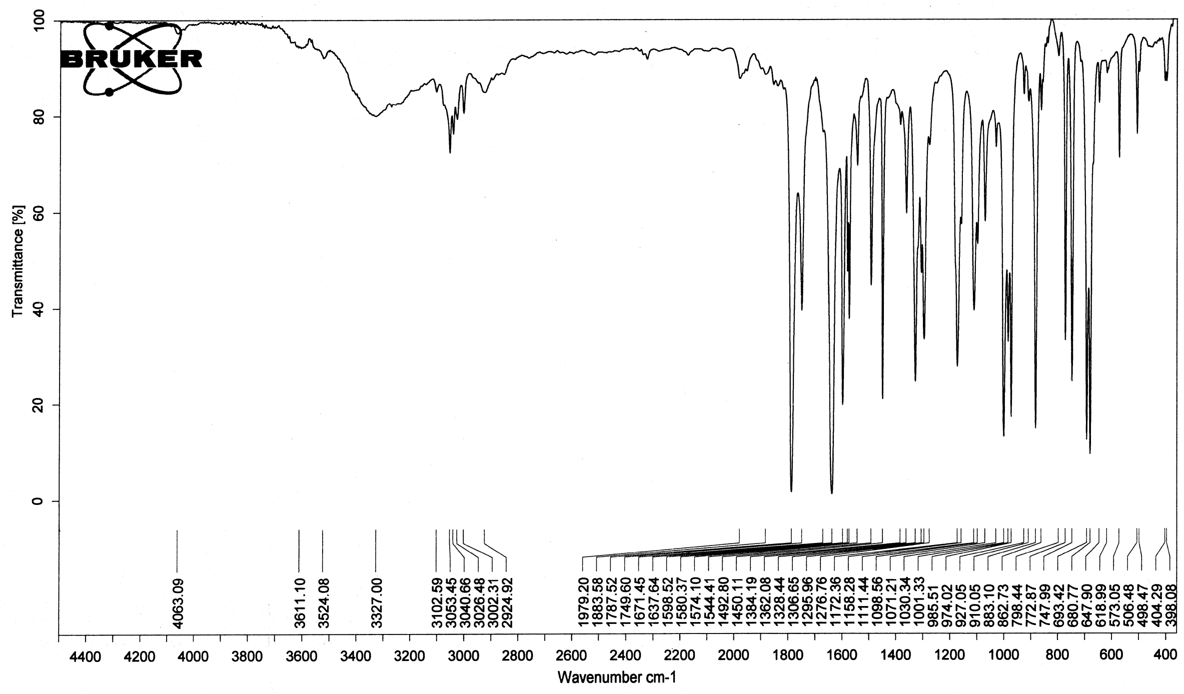

Enlarged spectrum in the range of 2000 - 400 cm^{-1}.

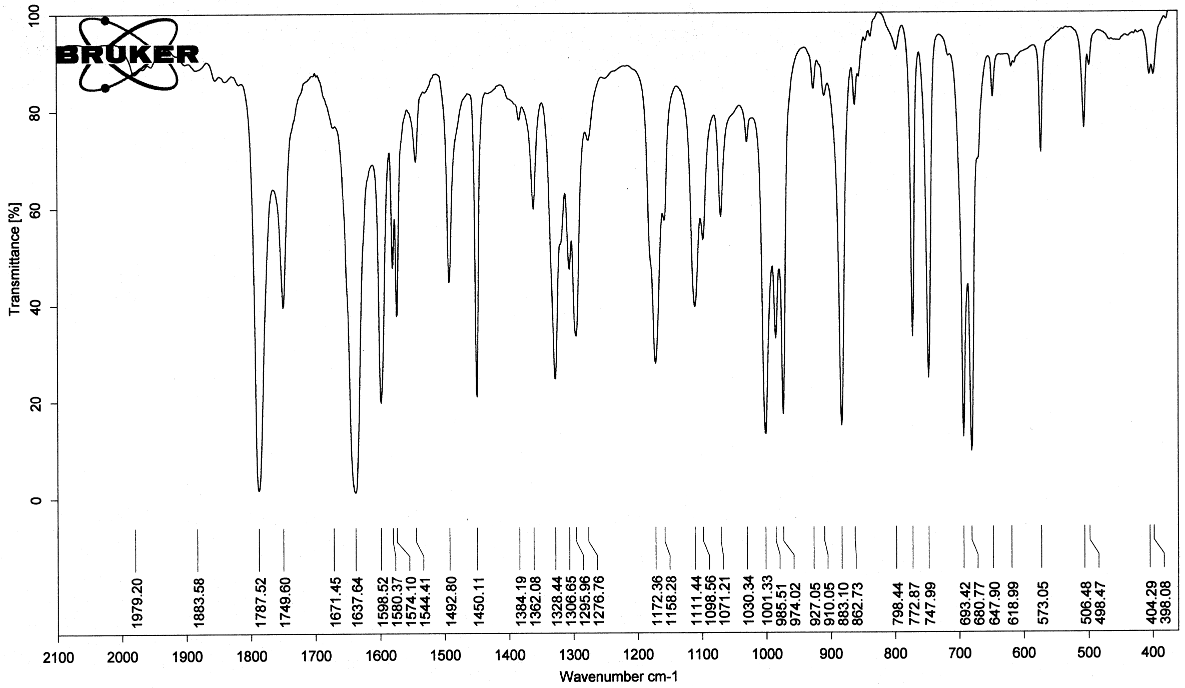

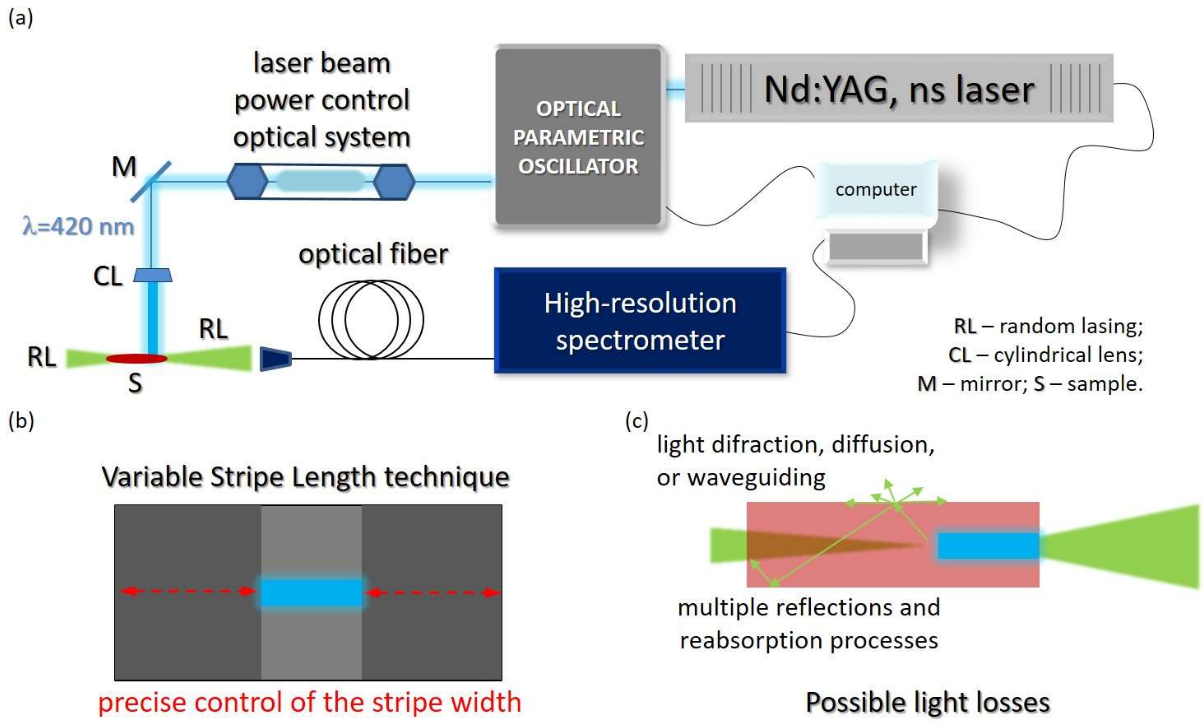

Figure S1. Light amplification setup (a) with technical details related to the VSL technique (b) and possible light diffusion (losses) mechanisms (c).

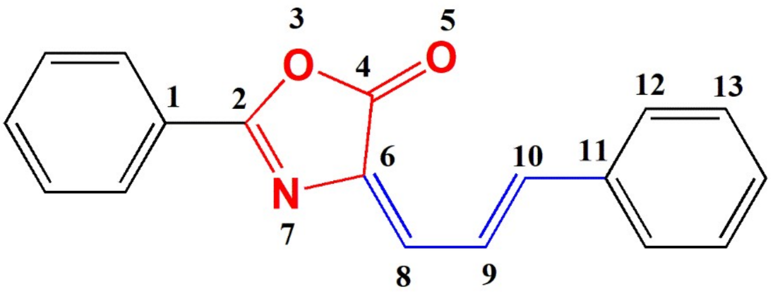

Figure S2. Atom numbering scheme.

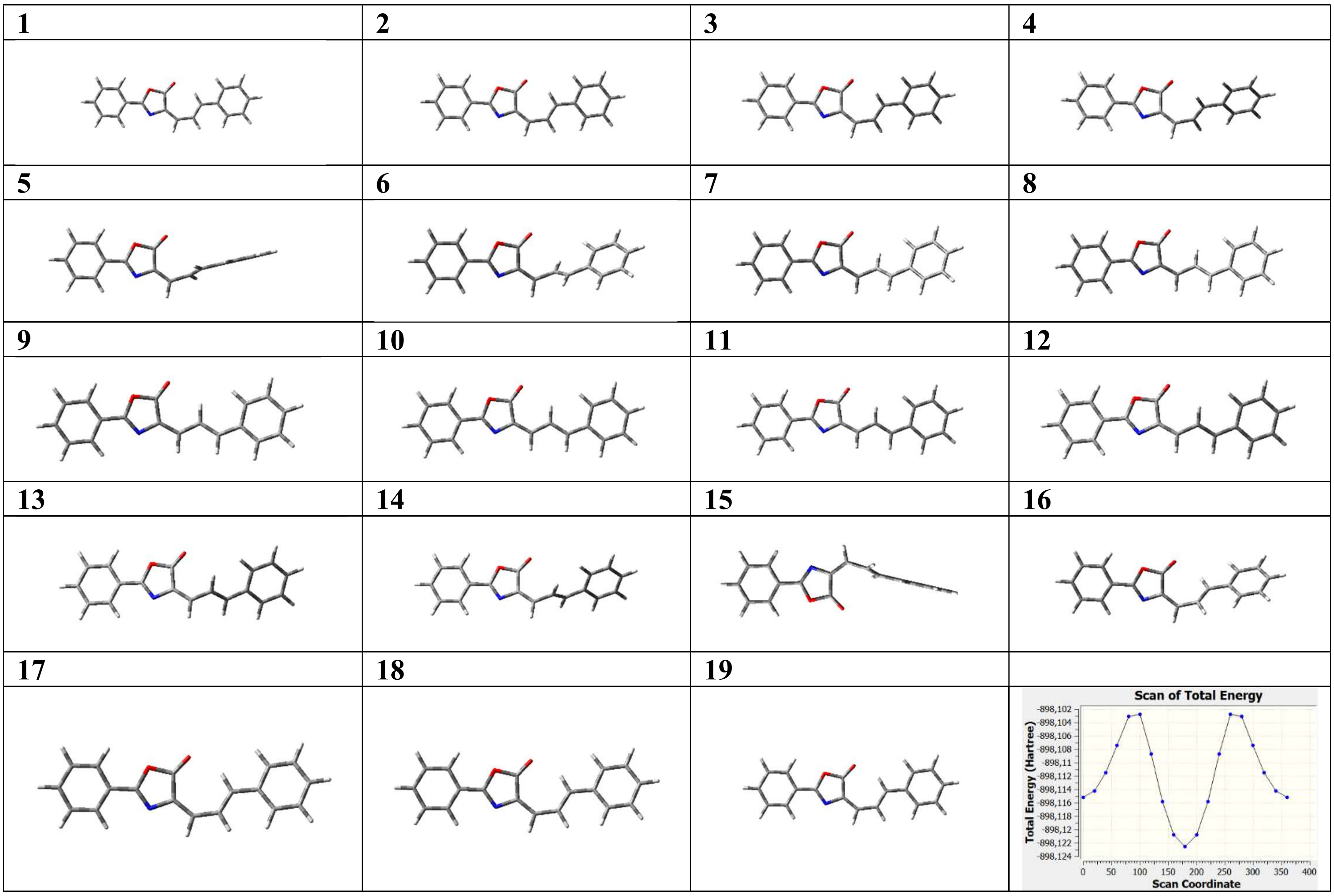

Figure S3. Results of a Potential Energy Surface (PES) scan for dihedral angle C6=C8-C9=C10 (wB97xd/6-33++G(d,p)).

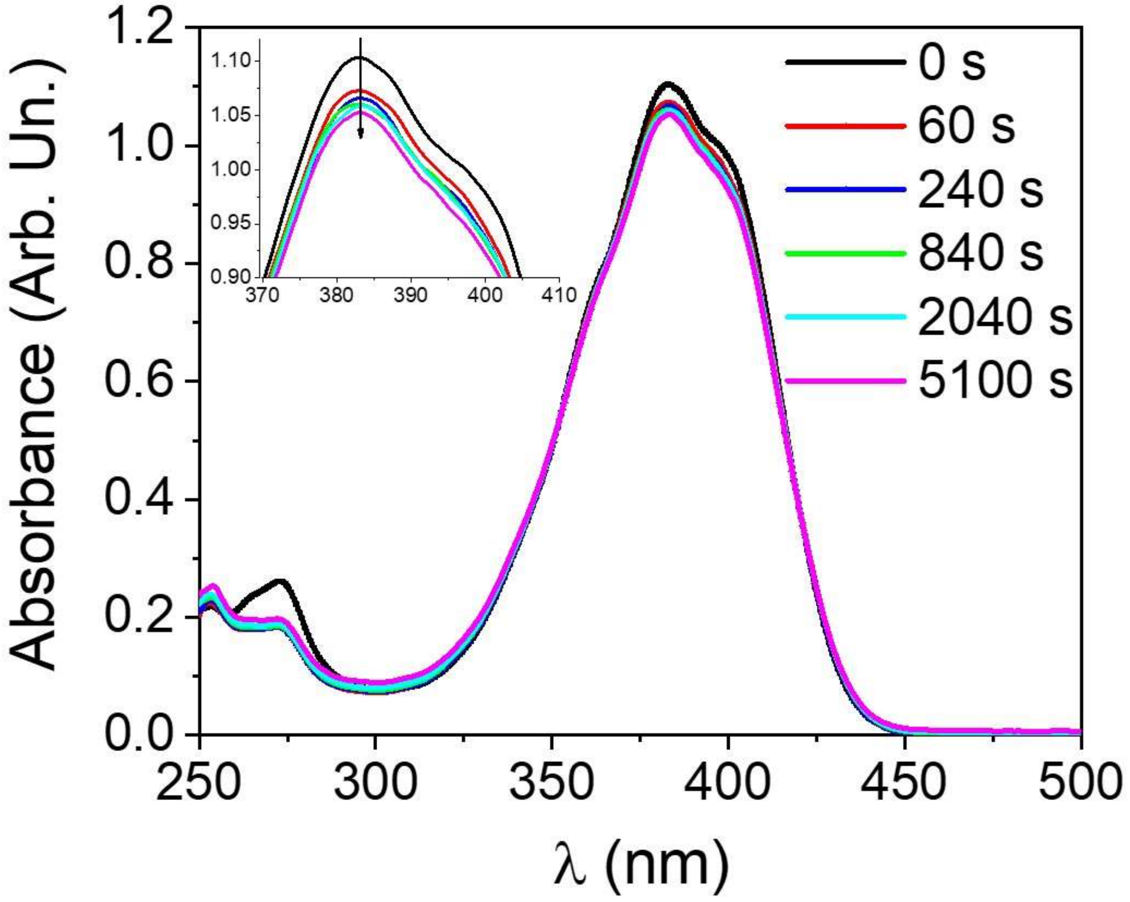

Figure S4. Time-dependent changes in absorption spectra of Ox-π,π-Ph in EtOAc after irradiation with DPSS laser (408 nm) at a light intensity of 35 mW. Dye concentration was 1.78×10^{-5} M.

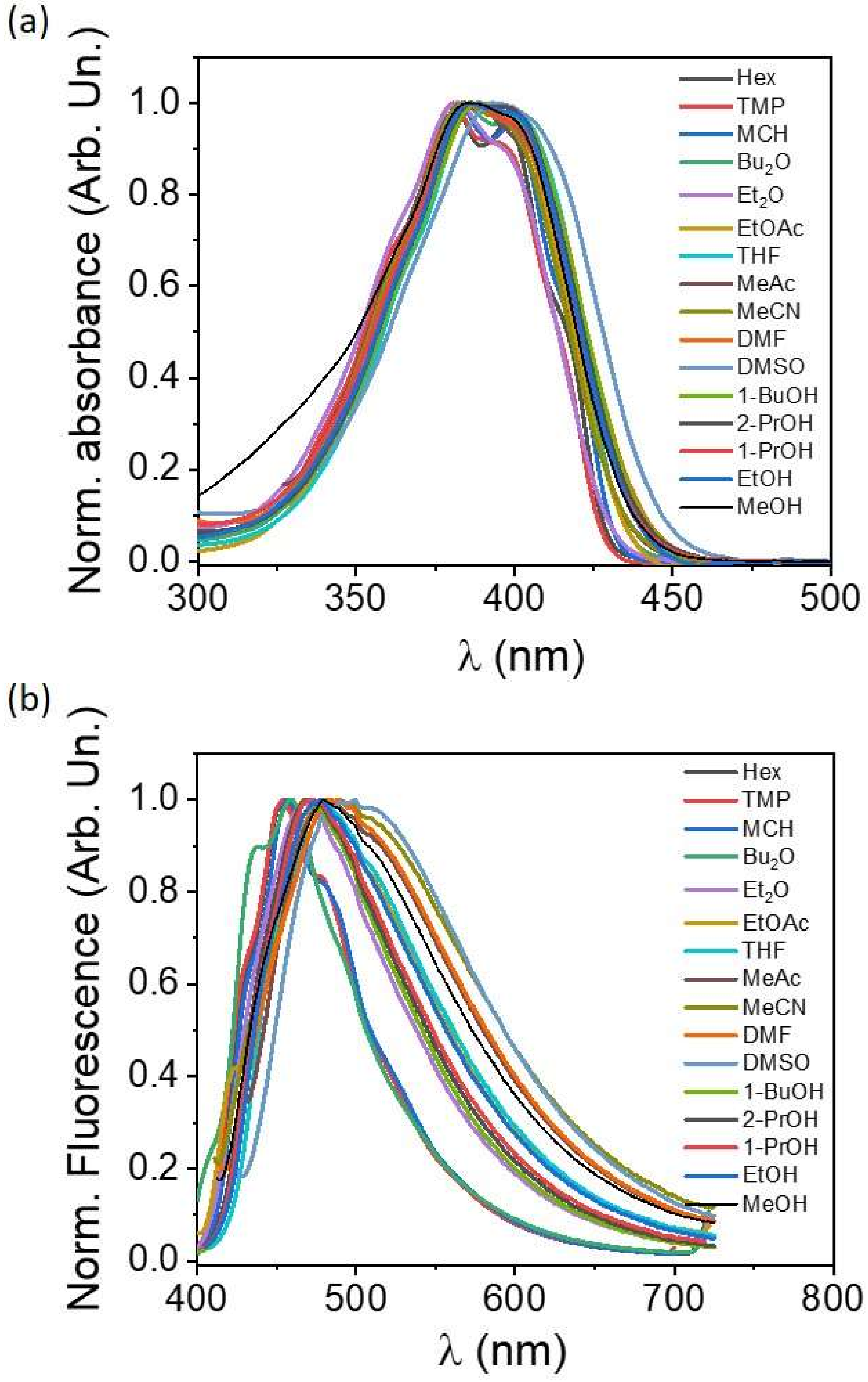

Figure S5. The normalized electronic absorption (a) and fluorescence (b) spectra of Ox-π,π-Ph in solvents of different polarities.

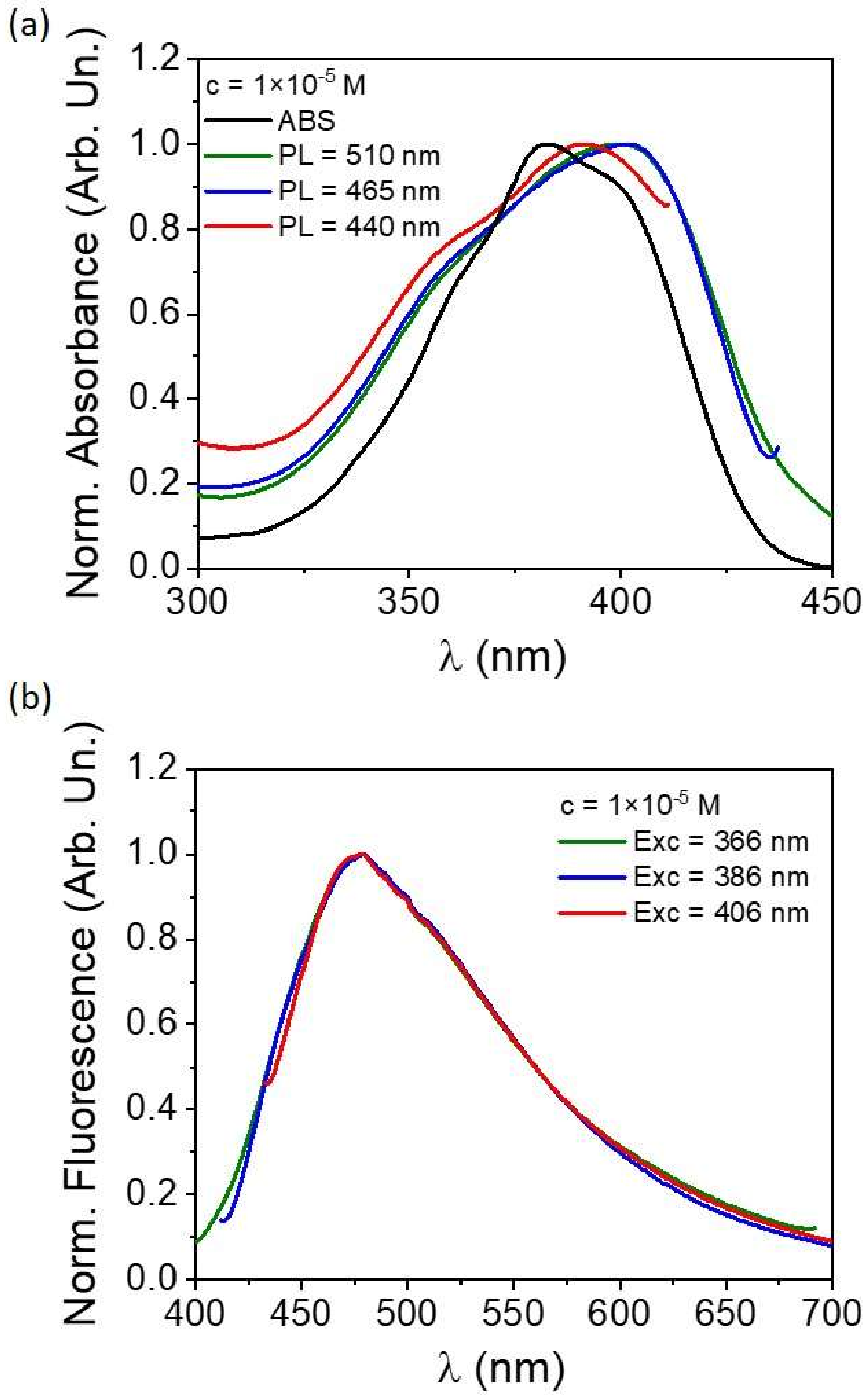

Figure S6. Normalized fluorescence excitation (a) and fluorescence (b) spectra of Ox-π,π-Ph in EtOAc recorded for different observation and emission wavelengths.

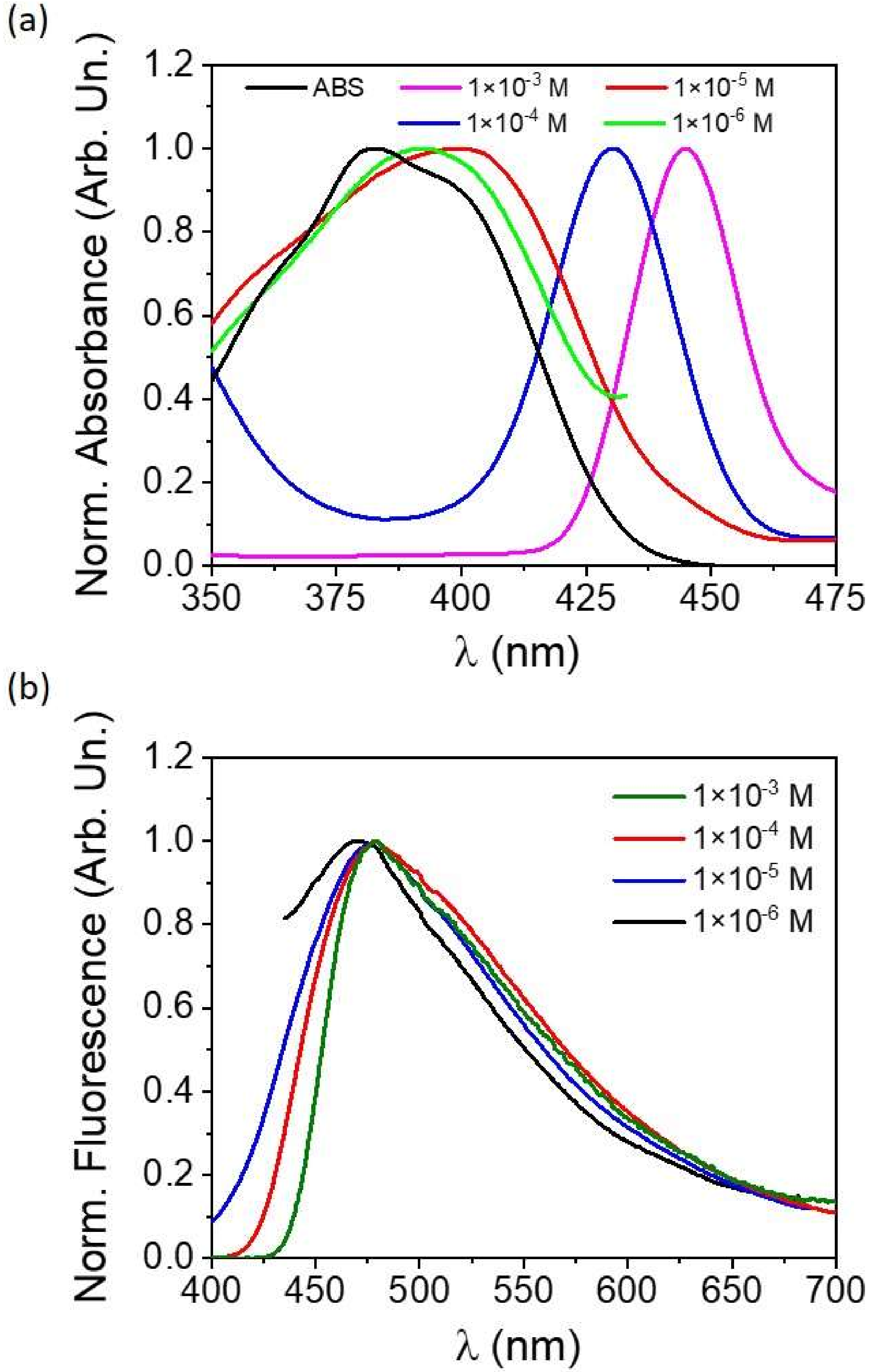

Figure S7. Normalized (a) fluorescence excitation (λ_{EM} = 510 nm) and (b) fluorescence (λ_{EX} = 366 nm) spectra of Ox-π,π-Ph in EtOAc recorded for solution of different concentration.

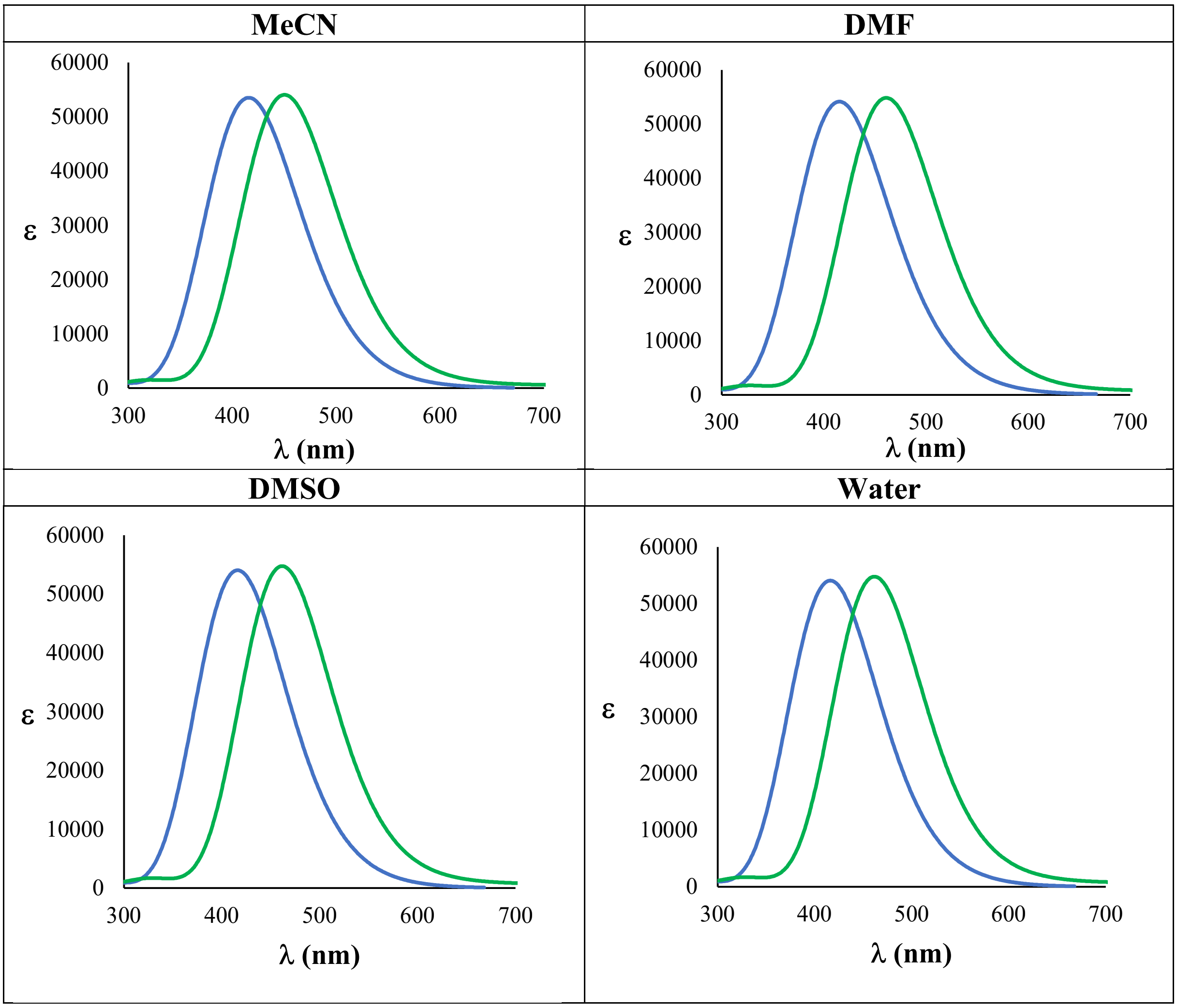

Figure S8. Comparison of the theoretical absorption maxima bands determined for monomer and dimer in different solvents.

Table S1. Solvent parameters and values of solvent polarity functions according to *Catalán*.

Solvent	Type[a)]	ε	n_{20}^{D}	E_T^N	SP	SdP	SA	SB
n-Hexane, Hex	NHB	1.8863	1.37486	0.009	0.616	0	0	0.056
2,2,4-TriMethylPentane, TMP	NHB	1.94	1.39145	n.d.	0.618	0	0	0.044
MethylCycloHexane, MCH	NHB	2.02	1.42312	n.d.	0.675	0	0	0.069
DiButyl Ether, Bu_2O	HBA	3.10	1.3992	0.071	0.672	0.175	0	0.637
DiEthyl Ether, Et_2O	HBA	4.335	1.35243	0.117	0.617	0.385	0	0.562
EthylAcetate, EtOAc	HBA	6.053	1.37239	0.099	0.656	0.603	0	0.542
TetraHydroFuran, THF	HBA	7.58	1.40716	0.207	0.714	0.634	-0.012	0.591
Aceton, MeAc	HBA	20.70	1.35868	0.355	0.651	0.907	0.007	0.475
Acetonitryl, MeCN	HBA-HBD	35.94	1.34411	0.46	0.645	0.974	0.044	0.286
N,N-DiMethylFormamide, DMF	HBA	36.71	1.43047	0.404	0.759	0.977	0.031	0.613
DiMethylSulfoxide, DMSO	HBA	46.45	1.4793	0.444	0.83	1	0.072	0.647
1-Butanol, 1-BuOH	HBA-D	17.51	1.3993	0.602	0.674	0.655	0.341	0.809
2-Propanol, 2-PrOH	HBA-D	19.92	1.3772	0.546	0.633	0.808	0.283	0.83
1-Propanol, 1-PrOH	HBA-D	20.45	1.38556	0.617	0.658	0.748	0.367	0.782
Ethanol, EtOH	HBA-D	24.55	1.36143	0.654	0.633	0.7830	0.400	0.6580
Methanol, MeOH	HBA-D	32.66	1.3284	0.762	0.608	0.904	0.605	0.545

[a)] NHB = non-hydrogen-bonding solvent; HBA = hydrogen bond acceptor; HBD = hydrogen bond donor; HBA-D = amphiprotic hydrogen bond acceptor-donor.

Table S2. Selected structural parameters of the *E* isomer in the ground state.

	GP	TMP	MCH	Bu_2O	Et_2O	THF	MeAc	MeCN	DMF	DMSO	Water
**bond lengths**											
C1-C2	1.45223	1.45216	1.45216	1.45213	1.45210	1.45202	1.45199	1.45198	1.45198	1.45198	1.45198
C4-O5	1.19838	1.20013	1.20023	1.20106	1.20159	1.20226	1.20290	1.20306	1.20307	1.20307	1.20138
C6-C8	1.36482	1.36542	1.36545	1.36575	1.36594	1.36610	1.36632	1.36638	1.36638	1.36638	1.36642
C8-C9	1.43060	1.43020	1.43018	1.43000	1.42989	1.42971	1.42959	1.42956	1.42956	1.42956	1.42954
C9-C10	1.35618	1.35672	1.35675	1.35701	1.35717	1.35738	1.35757	1.35762	1.35762	1.35762	1.35766
C10-C11	1.45339	1.45327	1.45326	1.45322	1.45320	1.45319	1.45319	1.45319	1.45319	1.45319	1.45319
**bond angles**											
O3-C4-O5	121.22134	121.08626	121.07806	121.01033	120.96609	120.90015	120.84780	120.83420	120.83344	120.83344	120.82397
O5-C4-C6	134.35553	134.38458	134.38651	134.40280	134.41367	134.44612	134.45836	134.46142	134.46159	134.46159	134.46368
N7-C6-C8	121.15638	121.13932	121.13839	121.13085	121.12597	121.12908	121.12224	121.12039	121.12029	121.12029	121.11899
C6-C8-C9	133.56802	133.67832	133.68446	133.72957	133.75644	133.78891	133.81676	133.82379	133.82418	133.82418	133.82903
C8-C9-C10	128.41321	128.42169	128.42152	128.41775	128.41352	128.40902	128.40251	128.40101	128.40093	128.40093	128.39998
C9-C10-C11	126.00071	125.97236	125.97078	125.95735	125.94751	125.92927	125.91071	125.90490	125.90456	125.90456	125.90020
C10-C11-C12	118.01045	118.02016	118.02087	118.02786	118.03393	118.04181	118.05496	118.05909	118.05934	118.05934	118.06244
**dihedral angles**											
O5-C4-C6-C8	0.00000	-0.00030	-0.00029	0.00000	0.00000	-0.05570	-0.05248	-0.05179	-0.05175	-0.05175	-0.05130
C6-C8-C9-C10	0.00000	0.00000	0.00000	0.00000	0.00000	0.00210	0.00210	0.00211	0.00211	0.00211	0.00212
C8-C9-C10-C11	-180.00000	-180.00000	-180.00000	-180.00000	-180.00000	-179.99936	-179.99943	-179.99945	-179.99945	-179.99945	-179.99946
C9-C10-C11-C12	-180.00000	-180.00000	-180.00000	-180.00000	-180.00000	-180.00000	-180.00000	-180.00000	-180.00000	-180.00000	-180.00000

Table S3. Selected structural parameters of the *Z* isomer in the ground state.

	GP	TMP	MCH	Bu_2O	Et_2O	THF	MeAc	MeCN	DMF	DMSO	Water
**bond lengths**											
C1-C2	1.45370	1.45341	1.45339	1.45237	1.45316	1.45302	1.45287	1.45284	1.45283	1.45283	1.45280
C4-O5	1.19055	1.19248	1.19260	1.19361	1.19431	1.19528	1.19628	1.19655	1.19657	1.19657	1.19679
C6-C8	1.35325	1.35354	1.35356	1.35374	1.35387	1.35410	1.35436	1.35445	1.35445	1.35445	1.35454
C8-C9	1.44662	1.44601	1.44599	1.44585	1.44569	1.44538	1.44504	1.44493	1.44493	1.44493	1.44482
C9-C10	1.34717	1.34758	1.34761	1.34787	1.32810	1.34844	1.34882	1.34894	1.34894	1.34894	1.34905
C10-C11	1.46245	1.46240	1.46239	1.46229	1.46218	1.46201	1.46181	1.46174	1.46174	1.46714	1.46168
**bond angles**											
O3-C4-O5	121.80051	121.64430	121.63484	121.55669	121.50659	121.44668	121.38646	121.37019	121.36927	121.36927	121.35757
O5-C4-C6	134.28025	134.30358	134.30474	134.31336	134.31393	134.30586	134.29532	134.29197	134.29176	134.29176	134.28616
N7-C6-C8	123.14839	123.17830	123.17878	123.18097	123.17385	123.15870	123.13559	123.12631	123.12571	123.12571	123.11329
C6-C8-C9	129.31740	129.43034	129.44010	129.53169	129.62138	129.75618	129.91957	129.97266	129.97593	129.97593	130.02549
C8-C9-C10	130.12962	130.29617	130.31099	130.45605	130.59678	130.79790	131.02681	131.09952	131.10402	131.10402	131.16552
C9-C10-C11	129.24613	129.41797	129.43197	129.57209	129.70803	129.88935	130.07542	130.13251	130.13607	130.13607	130.17919
C10-C11-C12	122.43280	122.56555	122.57593	122.68067	122.77290	122.89183	123.00820	123.04163	123.04366	123.04366	123.06525
**dihedral angles**											
O5-C4-C6-C8	-2.06162	-2.10796	-2.11452	-2.17058	-2.20992	-2.23640	-2.26621	-2.28147	-2.28261	-2.28261	-2.29291
C6-C8-C9-C10	46.09732	46.56937	46.59686	46.85854	46.99188	47.00948	46.90225	46.85879	46.85610	46.85610	46.80729
C8-C9-C10-C11	7.45724	7.26790	7.25896	7.16266	7.10066	7.03896	6.97380	6.95453	6.95343	6.95343	6.94974
C9-C10-C11-C12	34.45623	33.33006	33.24327	32.40464	31.67246	30.77033	29.89084	29.62180	29.60505	29.60505	29.38880

Table S4. Density difference plots.

	Z	***E***
GP	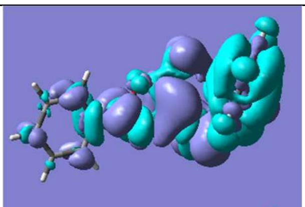	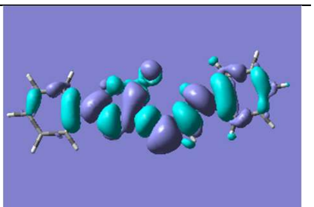
TMP	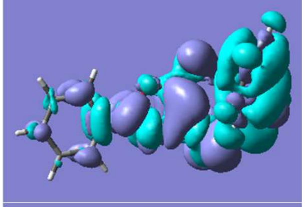	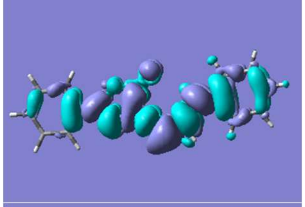
MCH	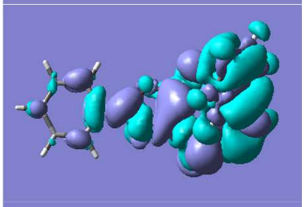	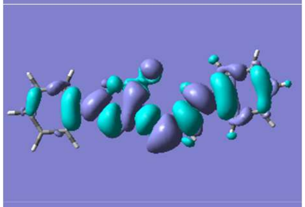
Bu_2O	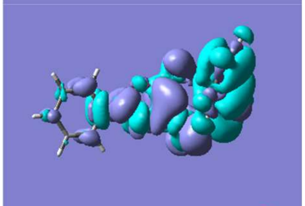	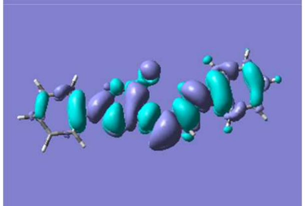
Et_2O	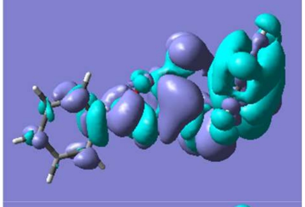	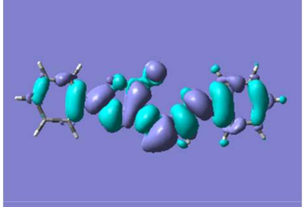
THF	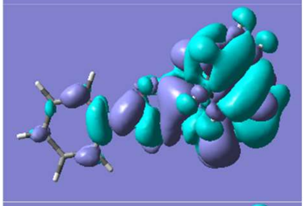	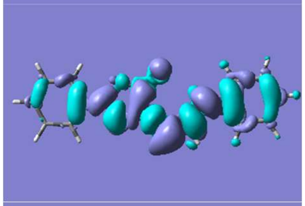
MeAc	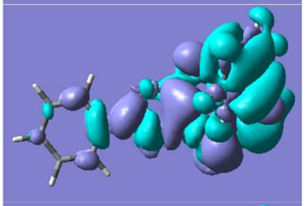	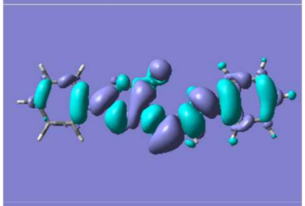
MeCN	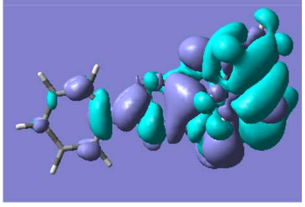	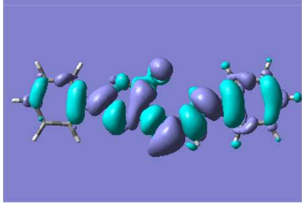

DMF	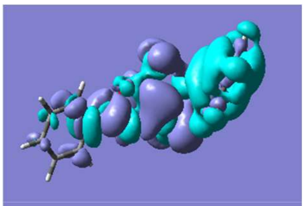	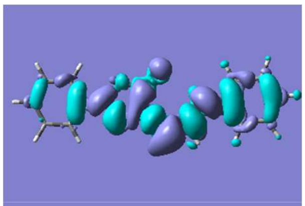
DMSO	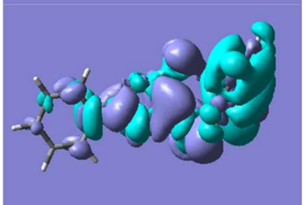	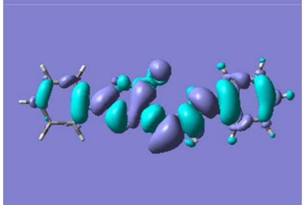
Water	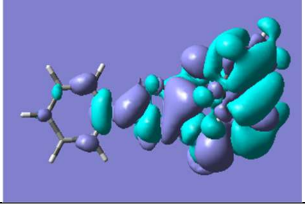	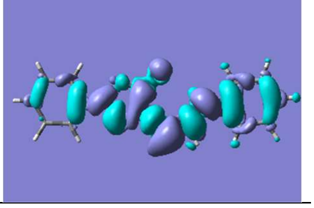

Table S5. The frontier orbital energies in different solvents. All values are given in eV.

***Z* isomer**	E_{HOMO}	E_{LUMO}	E_{GAP}	η	μ	χ
GP	-6.2712	-2.6011	3.6701	1.8350	-4.4361	4.4361
TMP	-6.3204	-2.6615	3.6589	1.8295	-4.4910	4.4910
MCH	-6.3237	-2.6656	3.6581	1.8290	-4.4946	4.4946
Bu_2O	-6.3531	-2.7015	3.6516	1.8258	-4.5273	4.5273
Et_2O	-6.3729	-2.7274	3.6456	1.8228	-4.5502	4.5502
THF	-6.3988	-2.7614	3.6374	1.8187	-4.5801	4.5801
MeAc	-6.4246	-2.7962	3.6284	1.8142	-4.6104	4.6104
MeCN	-6.4314	-2.8057	3.6257	1.8129	-4.6186	4.6186
DMF	-6.4320	-2.8063	3.6257	1.8129	-4.6191	4.6191
DMSO	-6.4320	-2.8063	3.6257	1.8129	-4.6191	4.6191
Water	-6.4366	-2.8134	3.6233	1.8116	-4.6250	4.6250
***E* isomer**	E_{HOMO}	E_{LUMO}	E_{GAP}	η	μ	χ
GP	-6.1073	-2.8585	3.2488	1.6244	-4.4829	4.4829
TMP	-6.1490	-2.9075	3.2414	1.6207	-4.5282	4.5282
MCH	-6.1520	-2.9111	3.2409	1.6204	-4.5315	4.5315
Bu_2O	-6.1784	-2.9407	3.2376	1.6188	-4.5595	4.5595
Et_2O	-6.1966	-2.9611	3.2355	1.6177	-4.5789	4.5789
THF	-6.2205	-2.9881	3.2325	1.6162	-4.6043	4.6043
MeAc	-6.2450	-3.0147	3.2303	1.6151	-4.6299	4.6299
MeCN	-6.2516	-3.0221	3.2295	1.6147	-4.6368	4.6368
DMF	-6.2521	-3.0224	3.2297	1.6149	-4.6372	4.6372
DMSO	-6.2521	-3.0224	3.2297	1.6149	-4.6372	4.6372
Water	-6.2567	-3.0275	3.2292	1.6146	-4.6421	4.6421

Table S6. CT parameters for the bright low-lying excited state of **Ox-π,π-Ph**.

	**Z**		**E**	
	q_{CT}	D_{CT}	q_{CT}	D_{CT}
GP	0.505	1.505	0.323	1.027
TMP	0.507	1.566	0.343	1.176
MCH	0.507	1.571	0.344	1.185
Bu_2O	0.509	1.616	0.347	1.200
Et_2O	0.509	1.652	0.348	1.191
THF	0.508	1.668	0.352	1.214
MeAc	0.506	1.682	0.354	1.214
MeCN	0.505	1.683	0.355	1.214
DMF	0.504	1.669	0.357	1.241
DMSO	0.504	1.673	0.357	1.239
Water	0.504	1.685	0.355	1.216

Table S7. Solvatochromic spectral parameters of Ox-π,π-Ph. The corresponding coefficients were calculated using multivariable linear regression applying *Catalán* approach. R = correlation coefficient. Polar solvents (1-BuOH, 1-PrOH, 2-PrOH, EtOH and MeOH) are excluded.

ν	ν_{ab}	ν_{fl}	$\Delta\nu^{SS}$
ν_0	27889±331	23302±342	4587±96
a_{SP}	-(2611±536) **62.4 %**	-(1992±553) **37.7%**	-(618±154) 26.5 %
b_{SdP}	-(233±92) 5.6 %	-(1324±95) **25.0 %**	1090±26 **46.7 %**
c_{SA}	1272±1528 30.4 %	1805±1576 34.2 %	-(533±440) 22.8 %
d_{SB}	-(70±139) 1.6 %	-(163±143) 3.1 %	93±40 4.0 %
R^2	0.918	0.988	0.998

Table S8. The vertical excitation energies (in nm).

	E		***Z***	
	λ_{Ab}	f	λ_{Ab}	f
GP	407.37	0.4320	391.97	1.2232
TMP	413.85	0.5074	401.36	1.3342
MCH	413.94	0.5118	401.64	1.3397
Bu_2O	414.46	0.5098	402.76	1.3346
Et_2O	414.77	0.5052	403.43	1.3253
THF	415.29	0.5166	404.76	1.3339
MeAc	415.64	0.5154	405.47	1.3244
MeCN	415.71	0.5151	405.64	1.3213
DMF	415.86	0.5273	406.08	1.3370
DMSO	415.85	0.5255	406.03	1.3346
Water	415.79	0.5153	405.79	1.3189

Table S9. The cLR corrected excitation energies (in nm).

	λ_{Ab}^{cLR}	
	Z	***E***
TMP	430.29	404.19
MCH	431.61	404.91
Bu_2O	431.22	405.54
Et_2O	429.63	405.45
THF	432.26	407.57
MeAc	430.74	407.63
MeCN	430.21	407.59
DMF	433.77	409.35
DMSO	433.25	409.08
Water	429.80	407.61

Table S10. The vertical de-excitation energies (in nm).

	λ_{FL}	
	Z	***E***
GP	491.63	487.08
TMP	528.11	457.80
MCH	530.32	457.17
Bu_2O	530.31	494.37
Et_2O	528.15	486.58
THF	532.93	482.95
MeAc	530.99	474.97
MeCN	530.25	473.05
DMF	536.20	476.35
DMSO	535.30	475.84
Water	529.68	475.15

**MeCN (λ_{Ab} = 445.21 nm)**	**DMF (λ_{Ab} = 448.77 nm)**
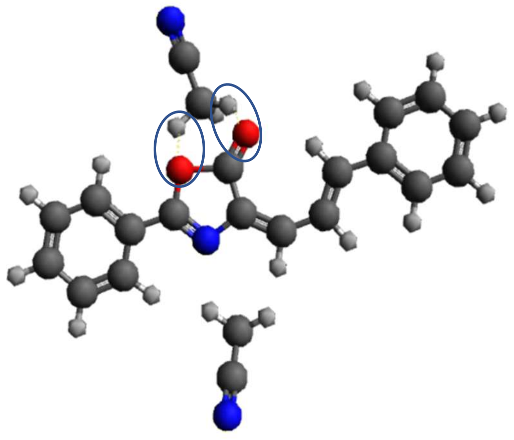	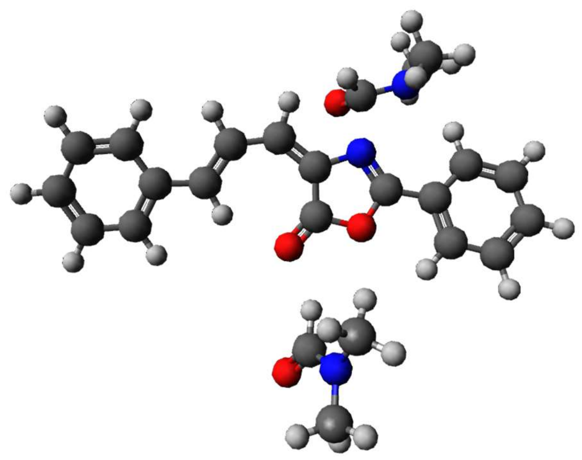
**DMSO (λ_{Ab} = 448.25 nm)**	**H_2O (λ_{Ab} = 444.80 nm)**
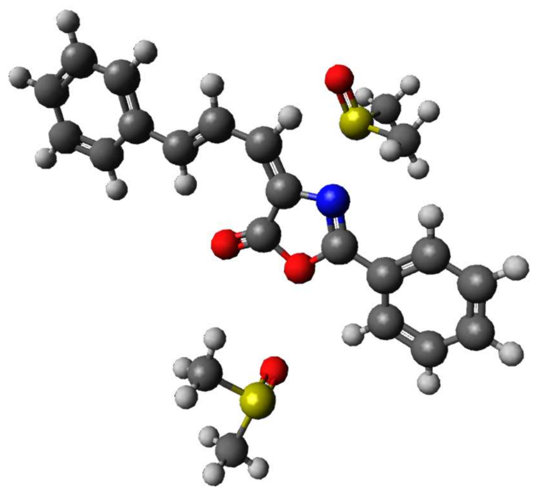	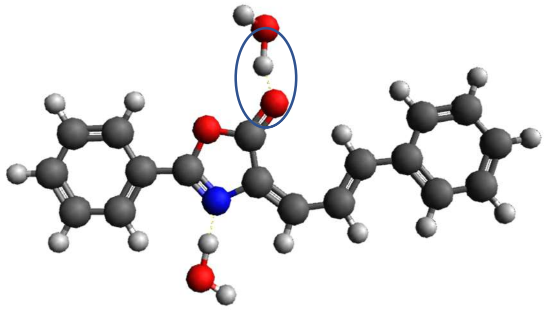

Figure S9. Interaction in the solvent-solute system in selected environments. Figures generated with Avogadro program.

Table S11. Calculated values of dipole moments (in D) for the ground and CT excited state.

	Z		***E***	
	μ_{GS}	μ_{CT}	μ_{GS}	μ_{CT}
GP	1.83	3.10	1.55	3.98
TMP	2.09	3.38	1.77	5.59
MCH	2.11	3.48	1.79	5.60
Bu_2O	2.25	3.68	1.91	5.77
Et_2O	2.35	4.95	1.99	5.84
THF	2.48	5.53	2.09	5.63
MeAc	2.63	5.50	2.20	5.70
MeCN	2.66	5.45	2.23	5.72
DMF	2.67	5.32	2.23	5.72
DMSO	2.68	5.33	2.24	5.73
Water	2.70	5.40	2.25	5.74

Table S12. Nonlinear properties of Ox-π,π-Ph isomers. Values are given in (a.u.).

	Z		***E***	
	α	β	α	β
GP	250.18	206.66	302.91	10.05
TMP	283.08	231.85	344.40	87.49
MCH	285.13	233.33	346.88	93.04
Bu_2O	302.38	247.78	367.46	141.67
Et_2O	314.32	260.72	381.08	175.43
THF	329.94	282.83	398.40	219.96
MeAc	346.16	313.24	415.74	260.95
MeCN	350.64	323.77	420.39	271.31
DMF	350.9	324.43	420.65	271.88
DMSO	352.05	324.62	421.90	273.71
Water	354.14	332.99	423.94	278.98
